# Evaluation of a micro-nutrient beverage mix intervention on biochemical parameters, growth, and strength in Indian children with diverse anthropometric profiles: An in-silico study

**DOI:** 10.1371/journal.pone.0318629

**Published:** 2025-08-25

**Authors:** Sahana Sringari, Surabhi Vijaykumar, Manali Sherkane, Nitika Vig, Megha Venkatesh, Suranjana Gupta, Karan Lomore, Venkatesh Kareenhalli

**Affiliations:** 1 MetFlux Research Private Limited, Bengaluru, India; 2 Mondelēz International Private Limited, Thane, India; 3 Indian Institute of Technology Bombay, Mumbai, India; Kerman University of Medical Sciences Physiology Research Center, IRAN, ISLAMIC REPUBLIC OF

## Abstract

**Introduction:**

Nutritional deficiencies in childhood can severely impact growth, making adequate micronutrient intake essential to prevent growth faltering. This study used an *in-silico* modeling framework to evaluate the impact of a micro-nutrient beverage (MNB) mix on growth, body composition, and strength in 7-to-9-year-old children within the 5th to 50th growth percentiles and diverse baseline anthropometries.

**Methods:**

The model was validated against national datasets (IAP 2015 and ICMR NIN 2024) before predicting the effects of a micro-nutrient beverage (MNB) mix on a virtual population of Indian children. The population was divided into three BMI types: Type 1 (low-normal height and weight), Type 2 (low height), and Type 3 (low height and weight), each reflecting specific nutrient deficiencies. Using a crossover study design for *in-silico* analysis, each BMI type underwent four experimental setups to assess the effects of the MNB mix with water (MNB-W) or milk (MNB-M). Key biochemical, anthropometric, and physical strength metrics were analyzed over simulated periods of 4, 8, and 12 months.

**Results:**

Both the MNB-W and MNB-M interventions significantly improved serum nutrient levels and growth parameters compared to the control group. All three BMI types exhibited notable increases in height and weight velocities, muscle mass, fat mass, and bone mineral content following the interventions. While BMI Type 3 children, who had major dietary inadequacies, showed the most pronounced improvements with the MNB-M intervention, children in BMI Types 1 and 2, with milder deficiencies, also experienced significant growth enhancements with both MNB-W and MNB-M interventions.Additionally, all BMI types showed improvements in physical strength with the MNB-M intervention, as seen in handgrip strength (HGS) and standing jump performance gains.

**Conclusion:**

This *in-silico* study emphasized the importance of baseline nutritional status in intervention effectiveness. Results underscore the importance of multiple micronutrient intervention, especially for children with severe deficiencies, in achieving optimal growth outcomes.

## Introduction

Hidden hunger, or micronutrient deficiency, is a widespread form of malnutrition affecting millions of children worldwide. This silent crisis is a global issue, driven by diets reliant on energy-dense but nutrient-poor foods, inadequate dietary diversity, and excessive processed food consumption [1]. Micronutrient deficiencies in childhood contribute to stunted growth, cognitive impairments, and long-term health risks, ultimately reducing individual potential and economic productivity [2,3]. In India, a substantial proportion of children continue to experience nutritional deficiencies, adversely affecting their growth trajectories and exacerbating the burden of malnutrition. [4,5]. Beyond caloric insufficiency, deficiencies in both growth and functional nutrients, which are essential for physical development and metabolic processes, are prevalent among children with moderate malnutrition, as indicated by their growth percentiles. These deficiencies contribute to diverse anthropometric profiles within the population, affecting parameters such as height, weight, and body composition [6–13]. A variation in nutritional status leads to the manifestation of different BMI types, where children may present with low height and weight for age, low height for age, or low weight for age, each contributing to distinct BMI percentiles. Children exhibiting low height and low weight for age, particularly in school-going populations, are at greater risk for adverse outcomes affecting their physical strength, cognitive development, and social interactions. Such deficits can compromise overall quality of life and limit future potential [14,15]. Tackling nutrient deficiencies, hence, remain a critical step to achieve optimum growth trajectories.

One effective strategy to address multiple nutrient deficiencies is through dietary interventions, such as nutrient enrichment of commonly consumed foods like milk. As a significant source of good-quality protein and key micronutrients, milk plays a vital role in tissue growth and repair, especially during critical periods of growth [16–18]. Enhancing the nutrient profile by adding essential nutrients to the milk, can further prove beneficial in improving bone health, muscle development, and physical strength in children with sub-optimal growth [19,20]. Clinical trials have shown that enriched milk not only addresses micronutrient deficiencies but also improves anthropometric and strength indices in children [21–23]. These findings highlight the potential of such nutritional interventions in optimizing child growth, particularly among populations at risk of growth faltering. However, while the observed benefits are promising, evaluating the effects of these interventions through large-scale clinical trials can be resource-intensive and time-consuming.

To overcome these challenges, *in-silico* modelling presents a powerful alternative. This computational approach allows researchers to simulate and assess the potential impact of nutritional interventions without the immediate need for extensive clinical trials. By using *in-silico* models, it is possible to predict the effects of nutrient interventions, on nutritional status, growth trajectories, and other growth-related outcomes, such as physical strength. This approach not only allows for the preliminary evaluation of intervention strategies but also enables the fine-tuning of intervention plans to maximize their efficacy before implementation in real-world settings.

The present study employs *in-silico* modelling to evaluate the effects of a micro-nutrient beverage (MNB) mix on the physical growth and strength outcomes of Indian children aged 7–9 years. Key nutrient deficiencies, highlighted in national surveys conducted on Indian children [24,25], are central to this analysis. The *in-silico* model simulates the impact of these deficiencies on anthropometric measures, categorizing children by BMI percentiles to understand how different nutrient deficiencies may influence growth outcomes. By predicting the potential effects of the MNB mix, particularly its role in enhancing physical strength and improving overall nutritional status, this study aims to assess the variability in responses among different BMI types categorized in this research.

## Materials and methods

### Population generation

An *in-silico* Indian child population between the 5th and 50th growth percentiles was generated by defining age, gender, height, body weight, and body fat percentage (%BF). While the baseline height and weight growth trajectories were benchmarked against IAP 2015 data, initial body composition and strength parameters were set using correlation equations. % BF, which is used as an input, was further used to estimate fat mass and lean mass [26]. Bone mineral content (BMC) was calculated based on a correlation with weight [27]. Handgrip strength (HGS) test and standing jump test performance were used as indicators of upper and lower body muscle strength, which was predicted using a correlation with fat-free mass [28,29].

### Modelling framework

The study employed an energetics-based framework to simulate the effects of the MNB mix on a child and extended it to model population-level outcomes (S1 Fig). The basal metabolic rate (BMR) of a child was calculated to align with the growth percentiles specific to each age and gender, such that a similar growth percentile is obtained in the following year. The total energy expenditure (TEE) of a child was assessed by the obtained BMR, macronutrient dependent thermic effect of food (TEF), and physical activity levels (PAL), as outlined in the following equation 1. The macronutrient composition was assumed to be 67% carbohydrates, 25% fat and 8% protein based on the known dietary compositions of Indian children [30,31]. The average physical activity level (PAL) was set in the range of 1.4–1.67, as per ICMR-NIN recommendations [32].


TEE=TEF+(BMR*PAL) (1)

Energy for growth was derived from the residual energy after subtracting the TEE from the total energy intake. The input net energy intake levels were estimated by adjusting for body weight, in accordance with the 2020 Recommended Dietary Allowances (RDA) for children [32] and the energy balance was calculated on a per-day basis. The residual energy, or energy allocated for growth, was used to calculate the daily increase in fat mass (ΔFM) and fat-free mass (ΔFFM), determined by the distribution coefficient ‘p’, which subsequently contributed to the overall weight gain. The estimated BMC [27] was used to obtain and further used to estimate lean mass from body weight and fat mass. The correlation equation used to predict physical strength from fat-free mass composition is presented in Equation 2, with an R² value of 0.8, indicating a strong correlation between handgrip strength and fat-free mass (kg). To validate this equation, we utilized independent clinical studies, as illustrated in S3 Fig.


Y=0.5693x+0.1562 (2)

### Micronutrient role linked to the energetics model

**Fig 1:**
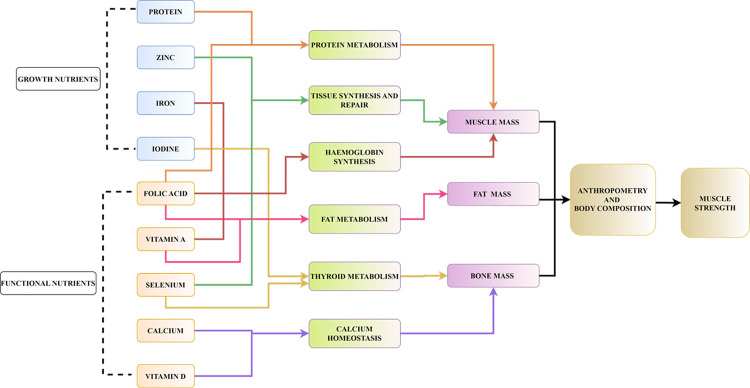
Association of micronutrients with physical growth and strength outcomes based on published studies.

To the above explained energy dependent model, micronutrient functionality was integrated to capture the variations in growth. The *in-silico* population was made deficient in eight common micronutrients namely, iron, zinc, calcium, vitamin D, folic acid, vitamin A, iodine, and selenium, as highlighted in the Indian surveys conducted on children [24,25]. Based on the established roles of these micronutrients (Fig 1), deficiencies of growth nutrients like protein, zinc, iodine, selenium, and iron were associated with low height for age. Similarly, deficiencies of functional nutrients such as vitamin A, folic acid, calcium, and vitamin D were associated with suboptimal growth of body tissues thereby affecting body composition. The degree of deficiency determined to the extent of growth faltering. To translate these known nutrient deficiency effects into the modelling framework, a set of correction factors denoted as epsilon (Ɛ), beta (β), eta (ƞ), and lambda (λ) were introduced, which were a function of percentage RDA (%RDA) intake of the eight micronutrients mentioned earlier. These correction factors penalized healthy growth if nutrient intake falls below 75% of the RDA. Specifically, Ɛ was linked to the RDA intake of protein, zinc, folic acid, and vitamin A, influencing the fat mass and fat-free mass distribution through the distribution coefficient, ‘p’. β was used to estimate BMC, associated primarily with the intake of calcium and vitamin D. To simulate monthly physical changes, nutrient utilization efficiency, denoted by ƞ, was adjusted using a hill function to estimate weight, while λ was adjusted for linear growth relative to growth percentiles. ƞ and λ were further linked to the %RDA intake of protein, zinc, iodine, selenium, and iron, allowing the model to predict the impact of these micronutrients on nutrient utilization and linear growth, respectively.

Consequently, the multi-micronutrient deficiencies modelled in the population resulted in varying combinations of height and weight percentiles, which were categorized into distinct BMI types according to BMI percentile ranges. The degree of penalty imposed on each BMI types is depicted in S4 Fig. At baseline, individuals classified as BMI type 1 were mildly deficient in calcium, zinc, vitamin A, and folic acid, meeting only 61% of the RDA for calcium and zinc, and 45% for vitamin A and folic acid. These deficiencies were associated with slightly below-average height and weight for age in this BMI type. BMI type 2 individuals exhibited more pronounced deficiencies, particularly in iron (35% of RDA), zinc (35% of RDA), calcium (60% of RDA), vitamin A (35% of RDA), vitamin D (60% of RDA), and folic acid (60% of RDA), which contributed to lower height for age in this type. BMI type 3 individuals were severely deficient in zinc, iron, iodine, selenium, and vitamin A, meeting only 35% of the RDA for each of these nutrients. These severe deficiencies were linked to both low height for age and low weight for age in this BMI type.

With the above-mentioned initial conditions, the model was set to predict the effect of nutritional interventions, defined by their energy intake, macronutrient, and micronutrient composition, on anthropometric and body composition changes (height, weight, BMI, FM, MM, and BMC). All parameters were calibrated to align with the growth percentile data from the IAP 2015, originally covering ages 3–16 years and the 3rd to 97th percentiles. For this study, the focus was specifically restricted to children aged 7–9 years within the 5th to 50th percentiles.

### Biochemical profile modelling and dose-response predictions

To simulate a population’s biochemical profile, this study leveraged data on micronutrient deficiency prevalence from national surveys conducted in children [24], ensuring that real-world variations in serum micronutrient levels were reflected. This data was used to establish the baseline serum or urinary levels of nutrients for the *in-silico* population. Single and combined micronutrient supplementation trials in children were analyzed to assess variations in response to dosages over time in both healthy and deficient states. From these studies, rates of change in serum micronutrient concentrations per unit dose were calculated separately for healthy and deficient children, enabling evaluation of the intervention’s impact on both populations. For individuals with low baseline nutrient levels, the rate of change specific to deficient populations was initially applied. As serum levels improved and reached the 25th percentile of sufficiency, the rate of change for healthy individuals was introduced, with further adjustments using Hill’s equation at the 50th percentile to account for physiological saturation. The obtained setup was then used to simulate the impact of MNB mix on serum micronutrient levels for each child.

### Population characteristics

To simulate a representative population of Indian children, virtual individuals aged 7–9 years were generated using growth data from the IAP 2015 charts, ensuring the model accurately reflected the growth patterns and anthropometric profiles specific to this population. The study cohort reflecting 5th to 50th weight percentiles, was further divided into two subgroups: the 5th to 25th percentile and the 25th to 50th percentile. A near-equal gender ratio (1:1) was maintained within the age groups. Each subgroup comprised of children classified into specific BMI types. The anthropometric differences between the BMI type 1, BMI type 2 and BMI type 3 are specified in Table 1. A population of 2,000 individuals per age and gender group was defined, each with specific height and weight ranges. Each experimental group hence had a population of 60000 virtual children which accounts to a total study population of 240000, as net cohort size of 4 experimental groups designed.

**Table 1:** Initial set up of the virtual population.

	5th to 25th Percentile Population			25th to 50th Percentile Population	
	**BMI Type 1**	**BMI Type 2**	**BMI Type 3**	**BMI Type 1**	**BMI Type 2**
**Weight range (kg)**	16.71 - 24.11	17.56 - 24.43	14.56 - 22.65	18.80 - 27.85	18.80 - 27.85
**Height range (cm)**	106.09 - 126.89	106.68 - 121.52	103.77 - 129.29	112.75 - 136.26	106.22 - 126.17
**BMI range**	14.04 - 16.06	15.24 - 16.80	13.09 - 14.17	14.04 - 16.06	15.94 - 18.42
**BMI percentile**	25th - 50th	60th - 65^th^	3rd - 10th	25th - 50th	60th - 65th
**Energy (kcal)**	1120 - 1611	1177 – 1625	902 - 1518	1260 - 2021	1260 - 2005
**Macronutrient composition**	Carbohydrate: ~ 67%; Protein: ~ 8%; Fat: ~ 25%				

### Intervention

The *in-silico* analysis employed a crossover study design, with each BMI type undergoing four distinct experimental setups. The experimental setups were designed to evaluate both, isolated and combined effects of specific nutrients in the composition. The control group received regular diet without milk. The MNB-W group received 20 g MNB mix in water, twice daily that included both macronutrients and micronutrients (Table 2). In contrast, the MNB-WC group received the 20 g MNB mix containing only the macronutrient component given with water. The rationale of setting up the MNB-W and MNB-WC groups were to differentially examine the effect of micronutrients on the output parameters. The MNB-M group received the 20 g MNB mix added in 200 ml milk (min. 6% fat) twice daily. Each group received the intervention for 12 months. The nutrient composition of the milk used in this study was based on data from IFCT 2017 and commercially available milk in India [33].

**Table 2:** Nutrient composition of different interventions. (MNB-W: Micronutrient beverage mix in water; MNB-WC: Isolated macronutrients from the micronutrient beverage mix in water; MN-M: micronutrient beverage mix in milk).

Nutrients	MNB-W	MNB-WC	MNB-M
**Energy (Kcal)**	155	155	584
**Carbohydrate (gm)**	34.7	34.7	68.3
**Protein (gm)**	2.8	2.8	17.5
**Fat (gm)**	0.7	0.7	27
**Vitamin A (mcg)**	316	0	724.4
**Folic acid (mcg)**	30	0	64.3
**Vitamin D (mcg)**	7.5	0	14
**Iron (mg)**	9.2	0	9.8
**Calcium (mg)**	200	0	684
**Zinc (mg)**	3	0	4.2
**Iodine (mcg)**	45.2	0	45.2
**Selenium (mcg)**	7.6	0	13.4

### Data analysis

The *in-silico* population generation and analysis were conducted using MATLAB, while statistical analyses were performed in Python (version 3.12.3), assuming a normal data distribution. A p-value of <0.05 was considered statistically significant for comparisons between two groups. Independent t-tests were applied to compare parameters across experimental groups, and Cohen’s d was calculated to determine effect size. Cohen’s d values of <0.2 were interpreted as small effects, 0.2 to 0.8 as medium effects, and >0.8 as large effects.

## Results

### Model validation

**Fig 2:**
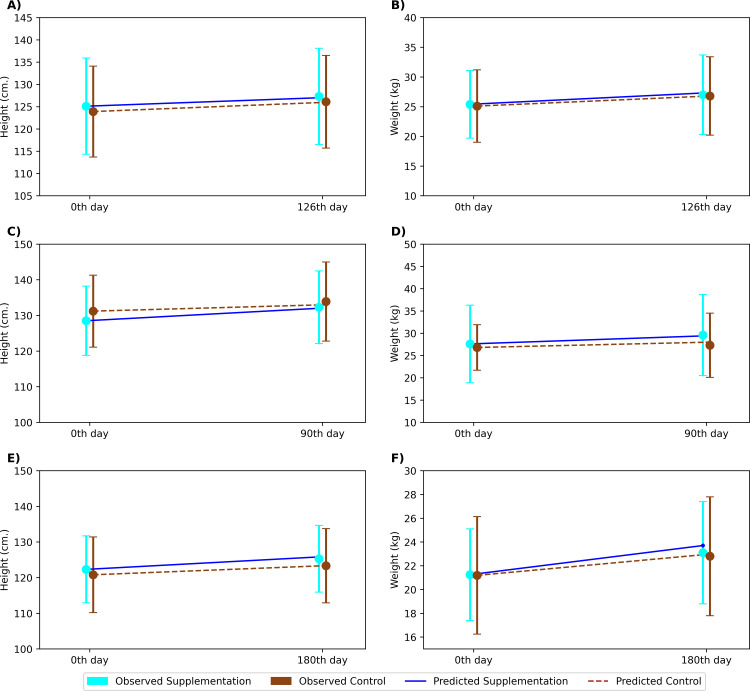
Validating predictions of intervention-led anthropometric changes using published studies. Circles represent mean values observed in the sample studies. **(A)** height change due to iron supplementation; **(B)** weight change due to iron supplementation; **(C)** height change due to zinc supplementation (BMI type 2); **(D)** weight change due to zinc supplementation (BMI type 2) **(E)** height change due to zinc supplementation (BMI type 3); **(F)** weight change due to zinc supplementation (BMI type 3). Blue circle: Control group; Brown circle: Supplementation group; Blue solid line: Prediction for control group; Brown dotted line: Prediction for intervention group.

The model was validated to predict changes in height and weight across different BMI types in response to an intervention, as illustrated in Fig 2. For example, cases of iron and zinc supplementation are presented, using Aguayo et al.‘s study on iron supplementation in anemic children as a validation sample [34]. (Figs 2A and 2B). The anthropometric characteristics of the population in this sample clinical trial align with the BMI type 1 population in the present study (34th weight percentile and 13th height percentile). In the trial, children aged 6–11 years received 3 mg/kg body weight of weekly iron supplementation for 126 days. Both the supplementation and control groups exhibited similar increases in height (2.2 cm) and weight (1.6 kg), indicating no significant impact of the intervention on anthropometry. When the model replicated the trial conditions, it predicted an average height increase of 2 cm (error <0.1%) in both groups, and weight increases of 1.9 kg in the supplementation group and 1.7 kg in the control group (error <0.1%). As the model linked iron to ƞ and λ correction factors, supplementation of iron could partially correct growth penalties on height and weight. However, deficiencies in other growth-related nutrients, such as protein, iodine, and zinc, were retained, which explain the lack of significant differences between the groups, consistent with the trial results.

Using two zinc supplementation studies, the model’s predictive capability was validated by assessing the effects of a single nutrient supplementation across two different BMI types defined in the study. Figs 2C and 2D depict a zinc supplementation trial conducted by Saldamli et al., which involved subjects with anthropometric characteristics similar to BMI type 2 (75th weight percentile and 23rd height percentile). In this trial, children with a mean age of 8 years received 2 mg/kg body weight of zinc supplementation for 90 days [35]. The trial reported a height increase of 3.8 cm in the supplementation group and 2.7 cm in the control group, while the model predicted increases of 3.5 cm and 1.8 cm, respectively. For weight, the trial observed increases of 2 kg in the supplementation group and 0.5 kg in the control group; the model predicted increases of 1.8 kg and 1.2 kg, respectively. The model’s predictions for mean height and weight changes showed an error margin of 0.2% to 2.5%. Further, Figs 2E and 2F represent validation of zinc supplementation effect on BMI type 3, which was done using a study published by Porto et. al., wherein, the study participants corresponded to the respective anthropometric ranges (11th weight percentile and 9th height percentile) [36]. In this trial, children with a mean age of 9 years received 5 mg/kg body weight of zinc supplementation for 365 days. The trial reported a height increase of 3 cm in the supplementation group and 2.5 cm in the control group, while the model predicted increases of 3.5 cm and 2.5 cm, respectively (error <0.5%). For weight, the trial observed increases of 1.8 kg in the supplementation group and 1.6 kg in the control group, while the model predicted increases of 2.5 kg and 1.8 kg, respectively (error <3%). The model could simulate height and weight increases in the supplementation groups due to zinc intake satisfying more than 75% of the RDA, correcting the imposed penalties by Ɛ, ƞ, and λ and thereby enhancing growth.

**Fig 3:**
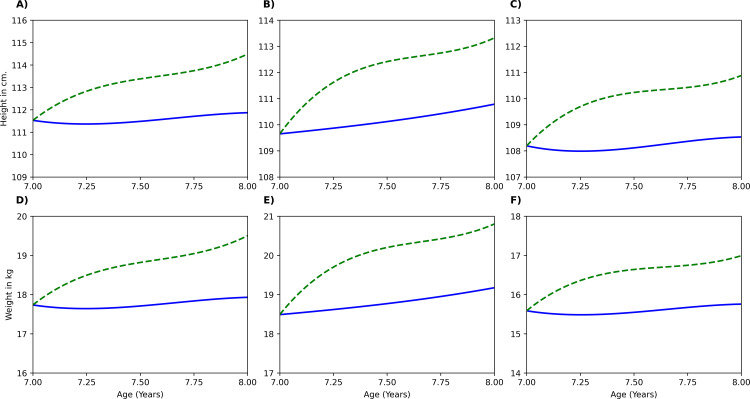
Model Simulation Case 1 – Effect of 10g daily protein supplementation in 7-year-old girls of three different BMI types: BMI type 1 (25th to 50th BMI percentile range; low-normal height and weight), BMI type 2 (60th to 65th BMI percentile range; low height), BMI type 3 (3rd to 10th BMI percentile range; low height and weight). Panels **(A)**, **(B)**, and **(C)** illustrate height trajectory of 3 girls belonging to BMI type1, type 2 and type 3 respectively; Panels **(D)**, **(E)**, and **(F)** illustrate weight trajectory of 3 girls belonging to BMI type1, type 2 and type 3 respectively. Blue solid line: Growth trajectory without intervention. Green dotted line: Growth trajectory with intervention.

**Fig 4:**
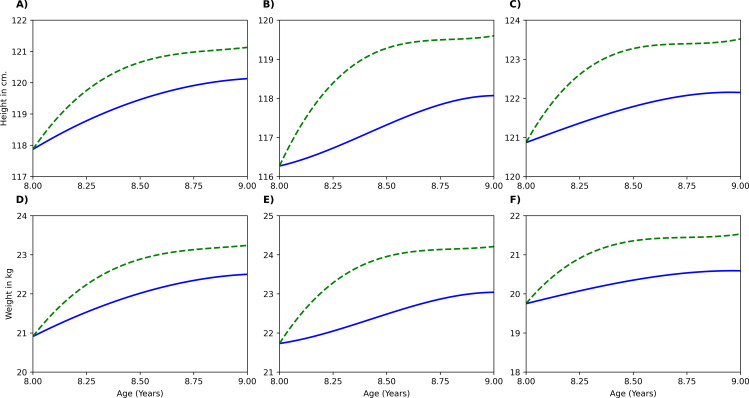
Model Simulation Case 2 – Effect of daily micronutrient – only supplementation achieving 100% RDA in 8-year-old boys of three different BMI types: BMI type 1 (25th to 50th BMI percentile range; low-normal height and weight), BMI type 2 (60th to 65th BMI percentile range; low height), BMI type 3 (3rd to 10th BMI percentile range; low height and weight). Panels **(A)**, **(B)**, and **(C)** illustrate height trajectory of 3 boys belonging to BMI type1, type 2 and type 3 respectively; Panels **(D)**, **(E)**, and **(F)** illustrate weight trajectory of 3 boys belonging to in BMI type1, type 2 and type 3 respectively. Blue solid line: Growth trajectory without intervention. Green dotted line: Growth trajectory with intervention.

Moreover, the model could also predict effect of a nutrient intervention over a specific dosage and duration. Example cases of protein and micronutrient supplementation are illustrated in Fig 3 and Fig 4 respectively. Fig 3 illustrates case 1, a protein intervention in which three 7-year-old girls with different weight and BMI percentiles received a daily supplement of 10 g for one year. The post intervention changes are shown for each BMI type: Type 1 (16th weight percentile and 31st BMI percentile), Type 2 (22nd weight percentile and 64th BMI percentile), Type 3 (11th weight percentile and 4th BMI percentile). Adequate protein intake could lead to a 3% increase in height and a 10% increase in weight compared to baseline across all BMI types. No significant differences between BMI types were observed as the basis of different BMI types were multi-micronutrient deficiencies. Similarly, Fig 4 depicts case 2, a scenario where an 8-year-old boy has 100% RDA intake of all key micronutrient intake. The post intervention changes in BMI type 1 (17th weight percentile and 36th BMI percentile), BMI type 2 (24th weight percentile and 63rd BMI percentile), BMI type 3 (5th weight percentile and 6th BMI percentile), are shown. Under control conditions, children in BMI types 1, 2, and 3 gained 2.26 cm, 1.8 cm, and 1.28 cm in height, respectively whereas with micronutrient intervention, an additional height gain of 1 cm, 1.5 cm, and 1.4 cm in BMI types 1, 2, and 3, respectively was observed. Similarly, the intervention led to additional weight gains of 0.74 g, 1.17 g, and 0.94 g in BMI types 1, 2, and 3 at the 12th month. Overall, the intervention resulted in a 2% increase in height and a 10% increase in weight across all BMI types compared to their respective baseline measurements. Thus, the model could monitor the growth trajectory of children and predict the possible growth outcomes due to an intervention. The overall error percentages in predicting all outcome parameters was within 5–20% (S2 Fig.).

### Assessing %RDA satisfied in the intervention groups

**Fig 5:**
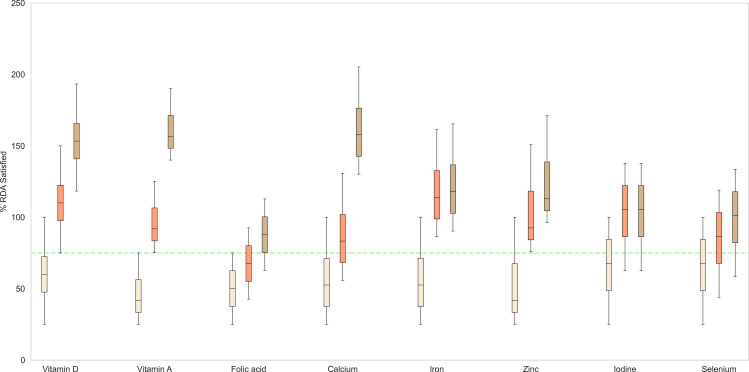
Percentage of RDA satisfied for various micronutrients across different intervention groups, given over and above the basal diet. Box: interquartile range; top, middle, and bottom edge of the box: Q1, Q2, Q3 respectively. White: Control; Orange: MNB-W; Brown: MNB-M, green dotted line: 75% RDA mark.

The MNB mix intervention was administered to the virtual population using both, water and milk as mediums, to the MNB-W and MNB-M groups respectively, and %RDA met in each group was subsequently assessed. Fig 5 shows the post-intervention comparison of the %RDA met for micronutrients in both the experimental groups. Compared to milk, the MNB mix intervention when supplemented over and above the basal diet could achieve greater nutrient adequacy in both the groups. However, variations corresponding to the baseline intake differences in BMI types were observed. The MNB-W group showed that individuals classified as BMI types 1 and 2 met more than 75% of the RDA for all assessed nutrients, except for folic acid, for which 65% of the RDA was achieved. In contrast, individuals classified as BMI type 3 met 75% of the RDA for most nutrients but fell short for iodine and selenium, achieving 73% and 54% of their respective RDAs.

In the MNB-M group, individuals classified as BMI types 1 and 2 could meet more than 75% of the RDA for all assessed nutrients. In contrast, those classified as BMI type 3 achieved 73% and 68% of the RDA for iodine and selenium, respectively, while meeting the 75% RDA threshold for the other nutrients.

### Effect of MNB mix on serum nutrient levels

The biochemical platform, developed using the aforementioned methodology, was validated using published nutrient intervention trials in children (S5 Fig). The predictions for all serum nutrient changes were made within 15% error. The platform was subsequently employed to predict serum nutrient changes under different intervention scenarios, using MNB-W and MNB-M groups for comparison.

As shown in S6.A Fig, the MNB-M intervention increased serum calcium at an average rate of 0.01 mg/dL per month over the 12-month period. While this change was statistically significant (p < 0.0001), the Cohen’s d value of 0.5 suggested a medium effect size (S3 Table). In S6.B Fig, the complete overlap between the MNB-W and MNB-M groups is attributed to the negligible iodine contribution from milk. As a result, both groups demonstrated mean urinary iodine levels within the reference range by the end of the intervention. Serum ferritin levels (S6.C Fig) increased at an average rate of 0.8 ug/dL throughout the intervention. In S6.D Fig, serum zinc levels showed the highest rate of change of 13.15 ug/dL by the 4th month, after which the increase slowed, reaching physiological saturation. Zinc increased by 5 ug/dL from months 4–8 and by 1.8 ug/dL from months 8–12. Similarly, for serum vitamin A (S6.E Fig), the rate of change was 1.65 ug/dL until the 4th month, slowing to 1.1 ug/dL between months 4 and 8, and further decreasing to 0.9 ug/dL between months 8 and 12. In S6.F Fig, serum vitamin D increased by an average of 5.7 nmol/L by the 4th month, with an additional increase of 1.5 nmol/L by the 8th month, and a further 0.5 nmol/L rise between months 8 and 12. S6.G Fig shows that serum folate increased at an average rate of 0.3 ng/mL until the 4th month, followed by a reduced rate of 0.2 ng/mL for both the 8th and 12th month. Finally, S6.H Fig reveals the rate of increase in serum selenium, which was 5.3 ug/L by the 4th month, 4.6 ug/L by the 8th month, and 4 ug/L by the 12th month. These findings indicate that while most serum micronutrient levels showed a rapid increase during the initial months of intervention, their rates of change slowed as they approached near sufficiency levels.

### Effect of MNB mix in water on physical growth outcomes

**Fig 6:**
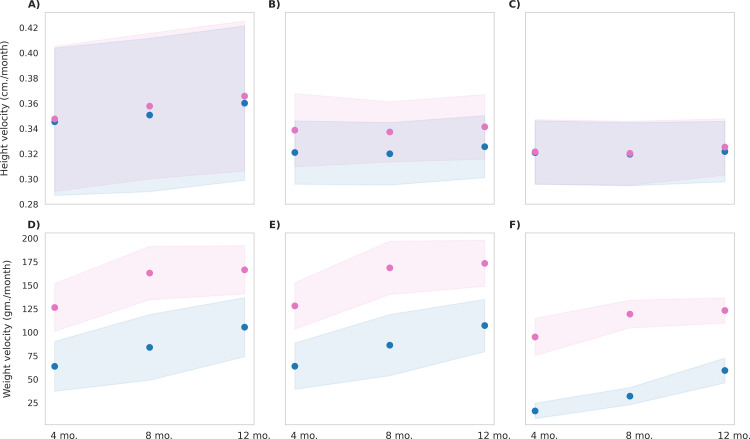
Post-intervention changes in anthropometric parameters among control group and MNB-W group. Blue represents the control group, and pink represents the MNB-W group. The mean values are indicated as circles and the shaded regions represent ±1σ, with a population size of 12,000 in each BMI type. **(A)** Height velocity in BMI Type 1 (25th to 50th BMI percentile range; low-normal height and weight), p < 0.001, observed, with a small effect size (Cohen’s d = 0.1). **(B)** Height velocity in BMI Type 2 (60th to 65th BMI percentile range; low height), p < 0.001, observed, with a large effect size (Cohen’s d = 0.6). **(C)** Height velocity in BMI Type 3 (3rd to 10th BMI percentile range; low height and weight), p < 0.001, observed, with a small effect size (Cohen’s d = 0.1). **(D)** Weight velocity in BMI Type 1 (25th to 50th BMI percentile range; low-normal height and weight), p < 0.001, observed, with a large effect size (Cohen’s d = 2.1). **(E)** Weight velocity in BMI Type 2 (60th to 65th BMI percentile range; low height), p < 0.001, observed, with a large effect size (Cohen’s d = 2.5). **(F)** Weight velocity in BMI Type 3 (3rd to 10th BMI percentile range; low height and weight), p < 0.001, observed, with a large effect size (Cohen’s d = 3.8).

The *in-silico* cohort was used to simulate the effect of the MNB mix intervention in water, where the intervention group included participants from all three BMI types. Fig 6 presents the post-intervention height and weight velocities, with population distributions represented by the shaded regions indicating standard error for each group. The figure highlights that, the basal diet taken by the control group was insufficient to support healthy growth. However, with the MNB-W intervention, average height velocities were 0.37, 0.34, and 0.33 cm/month for BMI types 1, 2, and 3, respectively. While all BMI types showed statistically significant improvement in height velocity (p < 0.001), the effect size was notably larger in BMI type 1 (Cohen’s d = 0.9) and BMI type 2 (Cohen’s d = 0.6) when compared to BMI type 3. Similarly, average mean weight velocities were 166, 173, 123 gm/month in BMI type 1, type 2, and type 3 respectively of the MNB-W group. The difference was statistically significant (p < 0.001) for all three BMI types with large effect size observed for BMI type 1 and 2 (Cohen’s d = 2.1 and 2.5 respectively), compared to BMI type 3.

**Fig 7:**
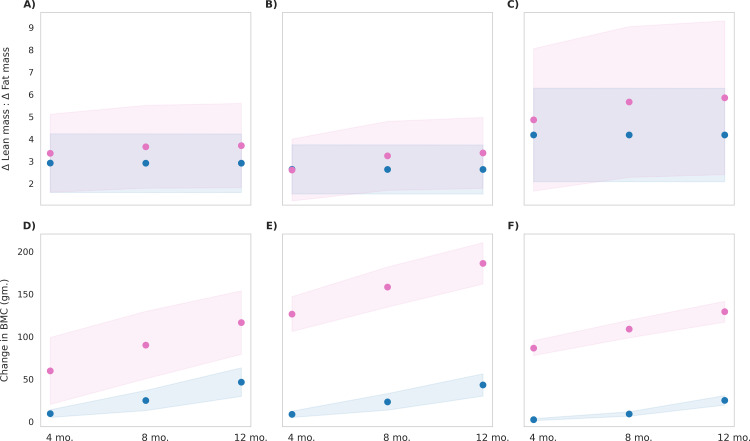
Post-intervention changes in body composition parameters among control group and MNB-W group. Blue represents the control group, and pink represents the MNB-W group. The mean values are indicated as circles and the shaded regions represent ±1σ, with a population size of 12,000 in each BMI type. **(A)** Delta lean mass to delta fat mass ratio in BMI Type 1 (25th to 50th BMI percentile range; low-normal height and weight), p < 0.001, observed, with a small effect size (Cohen’s d = 0.1) **(B)** Delta lean mass to delta fat mass ratio in BMI Type 2 (60th to 65th BMI percentile range; low height), p < 0.001, observed, with a small effect size (Cohen’s d = 0.1) **(C)** Delta lean mass to delta fat mass ratio in BMI Type 3 (3rd to 10th BMI percentile range; low height and weight), p < 0.001, observed, with a small effect size (Cohen’s d = 0.1) **(D)** Change in bone mineral content in BMI Type 1 (25th to 50th BMI percentile range; low-normal height and weight), p < 0.001, observed, with a large effect size (Cohen’s d = 2.3) **(E)** Change in bone mineral content in BMI Type 2 (60th to 65th BMI percentile range; low height), p < 0.001, observed, with a large effect size (Cohen’s d = 8.3) **(F)** Change in bone mineral content in BMI Type 3 (3rd to 10th BMI percentile range; low height and weight), p < 0.001, observed, with a large effect size (Cohen’s d = 14.3).

The MNB-W group exhibited a higher ratio of delta lean mass to delta fat mass (ΔLM/ΔFM) compared to the control group (Fig 7), primarily due to a greater increase in lean mass relative to fat mass. Among the BMI categories, children classified as BMI type 3 exhibited the highest increase in this ratio with the MNB-W intervention resulting in a gain of 600 grams in lean mass and no change in fat mass compared to the control group. In contrast, children in BMI type 1 and 2 experienced increases in fat mass of 100 grams each, while lean mass increased by 550 grams and 540 grams, respectively. The increase in lean mass demonstrated large effect sizes for BMI type 3, with Cohen’s d value of 0.8 and 0.9 when compared to BMI types 1 and 2, respectively. Conversely, the increase in fat mass observed in BMI types 1 and 2 resulted in effect sizes of 2.7 and 3.0, respectively, when compared to BMI type 3. Similarly, for BMC, BMI type 2 showed the greatest increase with the MNB-W intervention, resulting in a gain of 143 grams in BMC compared to the control group. Children in BMI type 1 and type 3 experienced increases of 70 grams and 104 grams in BMC, respectively, relative to the control group. This increase in BMC produced a large effect size of 2.5 for BMI type 2 compared to BMI type 3. The observed changes at the end of the intervention were statistically significant for all three BMI types when compared to the control group (p < 0.001). The results indicate that, among the BMI types, BMI type 1 and 2 showed most improvement in anthropometric indices while BMI type 3 exhibited improved body composition with MNB-W intervention.

While the discussed results pertain to the combined population within the 5th to 50th percentile range, detailed outcomes specific to the 5th to 25th percentile and the 25th to 50th percentile groups are provided in S4 Table and illustrated in Figs S8 through S19.

### Distinguishing contribution of micronutrients on physical growth outcomes

**Fig 8:**
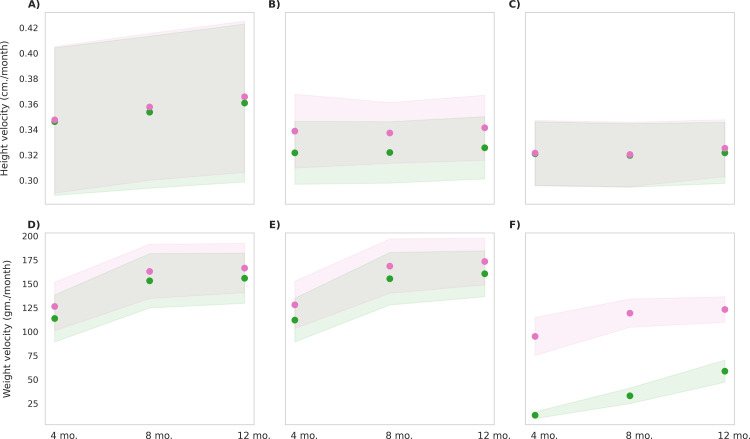
Post-intervention changes in anthropometric parameters among MNB-WC group and MNB-W group. Green represents the MNB-WC group, and pink represents the MNB-W group. The mean values are indicated as circles and the shaded regions represent ±1σ, with a population size of 12,000 in each BMI type **(A)** Height velocity in BMI Type 1 (25th to 50th BMI percentile range; low-normal height and weight), p < 0.001, observed, with a small effect size (Cohen’s d = 0.1) **(B)** Height velocity in BMI Type 2 (60th to 65th BMI percentile range; low height), p < 0.001, observed, with a large effect size (Cohen’s d = 0.6) **(C)** Height velocity in BMI Type 3 (3rd to 10th BMI percentile range; low height and weight), p < 0.001, observed, with a small effect size (Cohen’s d = 0.1) **(D)** Weight velocity in BMI Type 1 (25th to 50th BMI percentile range; low-normal height and weight), p < 0.001, observed, with a medium effect size (Cohen’s d = 0.4) **(E)** Weight velocity in BMI Type 2 (60th to 65th BMI percentile range; low height), p < 0.001, observed, with a medium effect size (Cohen’s d = 0.5) **(F)** Weight velocity in BMI Type 3 (3rd to 10th BMI percentile range; low height and weight), p < 0.001, observed, with a large effect size (Cohen’s d = 4.0).

The *in-silico* cohort was used to distinguish the contributions of macronutrients and micronutrients by establishing the MNB-WC group, which received only the macronutrient components of the MNB mix with water (Table 1). The intervention group included individuals belonging to all three BMI types. Fig 8 compare the post-intervention effects on anthropometric parameters between the MNB-WC and MNB-W group, highlighting the differential impact of macronutrient-only versus multi-micronutrient intervention. The MNB-W group demonstrated small but significant increase (p < 0.0001) in both height and weight velocities across all BMI types, likely due to the added effect of micronutrients. Specifically, height velocity in the MNB-W group versus the MNB-WC group was 0.37 vs. 0.36 cm/month, 0.34 vs. 0.33 cm/month, and 0.33 vs. 0.32 cm/month for BMI types 1, 2, and 3, respectively. The MNB-W intervention hence resulted in an additional height gain of 0.2 cm in the BMI type 2 population and 0.1 cm in the BMI type 1 population, with the height gain in BMI type 2 showing a medium effect size (Cohen’s d = 0.6). Similarly, weight velocity was higher in the MNB-W group compared to the MNB-WC group, with values of 166 vs. 156 g/month, 173 vs. 160 g/month, and 123 vs. 59 g/month for BMI types 1, 2, and 3, respectively. The weight gain in BMI type 3 yielded a large effect size (Cohen’s d = 4), while BMI types 1 and 2 achieved medium effect sizes (Cohen’s d = 0.4 and 0.5, respectively).

**Fig 9:**
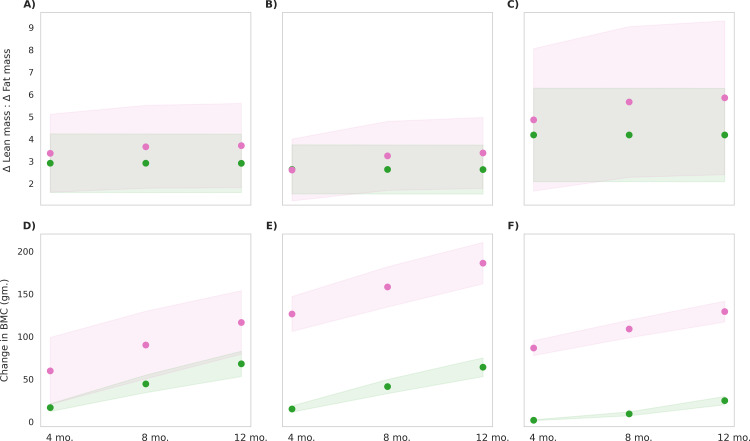
Post-intervention changes in body composition parameters among MNB-WC group and MNB-W group. Green represents the MNB-WC group, and pink represents the MNB-W group. The mean values are indicated as circles and the shaded regions represent ±1σ, with a population size of 12,000 in each BMI type. **(A)** Delta lean mass to delta fat mass ratio in BMI Type 1 (25th to 50th BMI percentile range; low-normal height and weight), p < 0.001, observed, with a small effect size (Cohen’s d = 0.1) **(B)** Delta lean mass to delta fat mass ratio in BMI Type 2 (60th to 65th BMI percentile range; low height), p < 0.001, observed, with a small effect size (Cohen’s d = 0.1) **(C)** Delta lean mass to delta fat mass ratio in BMI Type 3 (3rd to 10th BMI percentile range; low height and weight), p < 0.001, observed, with a small effect size (Cohen’s d = 0.1) **(D)** Change in bone mineral content in BMI Type 1 (25th to 50th BMI percentile range; low-normal height and weight), p < 0.001, observed, with a large effect size (Cohen’s d = 1.6) **(E)** Change in bone mineral content in BMI Type 2 (60th to 65th BMI percentile range; low height), p < 0.001, observed, with a large effect size (Cohen’s d = 7.4) **(F)** Change in bone mineral content in BMI Type 3 (3rd to 10th BMI percentile range; low height and weight), p < 0.001, observed, with a large effect size (Cohen’s d = 14.9).

The ratio of ΔLM/ΔFM was higher in the MNB-W group compared to the MNB-WC group, primarily due to a greater increase in lean mass relative to fat mass (Fig 9). Specifically, BMI types 1 and 2 in the MNB-W group gained 1.3 kg of lean mass, while BMI type 3 gained 500 g. In terms of fat mass increase, BMI type 2 gained highest fat mass (600 g), which was 100 g more than BMI type 1 while BMI type 3 showed an average fat mass gain of 100 g. This increased ΔLM/ΔFM ratio resulted in large effect sizes, with Cohen’s d values of 0.9 and 0.7 for BMI type 3 compared to types 2 and 1, respectively. Additionally, the MNB-W group exhibited a mean additional BMC increase of 48.6 g, 122.1 g, and 104.5 g in BMI types 1, 2, and 3, respectively, all corresponding to large effect sizes. The greater improvements in the MNB-W group, particularly in BMI type 2 and 3, can be attributed to the added effect of micronutrients in the MNB, enhancing both lean mass and BMC gains (S1 and S2 Tables).

### Effect of MNB mix with milk on physical growth outcomes

**Fig 10:**
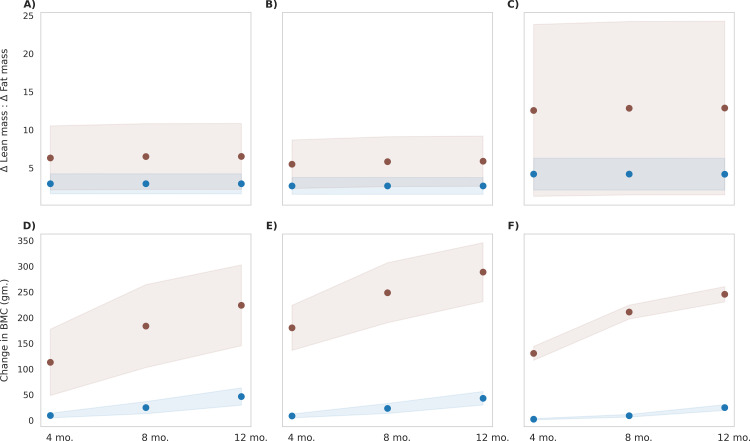
Post-intervention changes in body composition parameters among control group and MNB-M group. Blue represents the control group, and brown represents the MNB-M group. The mean values are indicated as circles and the shaded regions represent ±1σ, with a population size of 12,000 in each BMI type **(A)** Delta lean mass to delta fat mass ratio in BMI Type 1 (25th to 50th BMI percentile range; low-normal height and weight), p < 0.001, observed, with a small effect size (Cohen’s d = 0.1) **(B)** Delta lean mass to delta fat mass ratio in BMI Type 2 (60th to 65th BMI percentile range; low height), p < 0.001, observed, with a small effect size (Cohen’s d = 0.2) **(C)** Delta lean mass to delta fat mass ratio in BMI Type 3 (3rd to 10th BMI percentile range; low height and weight), p < 0.001, observed, with a medium effect size (Cohen’s d = 0.4) **(D)** Change in bone mineral content in BMI Type 1 (25th to 50th BMI percentile range; low-normal height and weight), p < 0.001, observed, with a large effect size (Cohen’s d = 3.8) **(E)** Change in bone mineral content in BMI Type 2 (60th to 65th BMI percentile range; low height), p < 0.001, observed, with a large effect size (Cohen’s d = 7.6) **(F)** Change in bone mineral content in BMI Type 3 (3rd to 10th BMI percentile range; low height and weight), p < 0.001, observed, with a large effect size (Cohen’s d = 21.3).

The *in-silico* cohort was further used to simulate the effects of the MNB mix intervention in milk, including participants from all three BMI types. Fig 10 presents the results comparing post-intervention body composition between the control and MNB-M group. The MNB-M intervention showed greatest impact in children classified as BMI type 3 population with 3.8 kg gain in lean mass. BMI type 1 and type 2 gained 3.4 kg and 3.2 kg respectively. The absolute fat mass gained in all BMI types 1 and 2 was 700 g on an average while BMI type 3 gained 500 g. Therefore, BMI type 3 exhibited a higher ratio of ΔLM/ΔFM yielding an effect size of 0.9 when compared to BMI type 1 and BMI type 2. Similarly, BMI type 3 gained higher BMC (220 g) by the end of intervention compared to BMI types 1 and 2, yielding a large effect size (Cohen’s d = 1.1 and 2.5, respectively). In context of total weight gain, the lean mass increase accounted for 79%, 78% and 86% of the total weight gained in BMI type 1, type 2, and type 3 respectively, suggesting a healthier weight gain.

Moreover, the MNB-M group exhibited the highest height and weight velocities in BMI type 3, with rates of 0.6 cm/month and 0.36 kg/month, respectively. In comparison, BMI type 1 showed height and weight velocities of 0.53 cm/month and 0.35 kg/month, while BMI type 2 demonstrated velocities of 0.52 cm/month and 0.34 kg/month (S7 Fig).

### Effect of MNB mix with milk on strength outcomes

**Fig 11:**
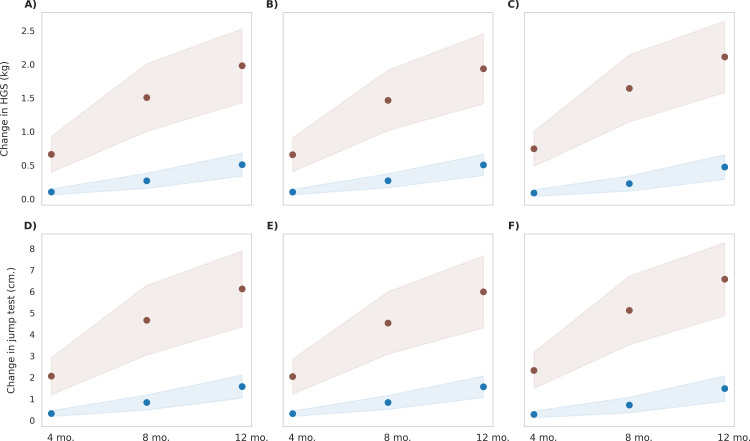
Post-intervention changes in strength parameters among control group and MNB-M group. Blue represents the control group, and pink represents the MNB-W group. The mean values are indicated as circles and the shaded regions represent ±1σ, with a population size of 12,000 in each BMI type. **(A)** Handgrip strength (HGS) test in BMI Type 1 (25th to 50th BMI percentile range; low-normal height and weight), p < 0.001, observed, with a large effect size (Cohen’s d = 6.6) **(B)** Handgrip strength (HGS) test in BMI Type 2 (60th to 65th BMI percentile range; low height), p < 0.001, observed, with a large effect size (Cohen’s d = 6.8) **(C)** Handgrip strength (HGS) test in BMI Type 3 (3rd to 10th BMI percentile range; low height and weight), p < 0.001, observed, with a large effect size (Cohen’s d = 7.3) **(D)** Change in standing jump test in BMI Type 1 (25th to 50th BMI percentile range; low-normal height and weight), p < 0.001, observed, with a large effect size (Cohen’s d = 6.3) **(E)** Change in standing jump test in BMI Type 2 (60th to 65th BMI percentile range; low height), p < 0.001, observed, with a large effect size (Cohen’s d = 6.5) **(F)** Change in standing jump test in BMI Type 3 (3rd to 10th BMI percentile range; low height and weight), p < 0.001, observed, with a large effect size (Cohen’s d = 7.0).

Fig 11 presents the simulation results comparing post-intervention effects on handgrip strength (HGS) and standing jump test performance between the control group and the MNB-M group. The MNB-M group demonstrated an average increase in HGS of 1.9 kg in the BMI type 1 and type 2 populations, and 2.1 kg in the BMI type 3 population. These improvements showed statistical significance (p < 0.0001) and corresponded to large effect sizes, with Cohen’s d values of 6.6, 6.8, and 7.3 for BMI types 1, 2, and 3, respectively. Additionally, standing jump performance significantly increased by 6 cm in BMI type 1 and 2 and 6.6 cm in BMI type 3, yielding effect sizes of 6.3, 6.5, and 7. The gains in HGS or standing jump performance demonstrated small effect sizes between the BMI types indicating that MNB-M intervention could lead to improved muscle strength in a child population with varying anthropometric profiles.

## Discussion

The present *in-silico* study highlights the potential of an energetics-based modelling framework to predict the impact of a MNB mix intervention on biochemical parameters, growth, and strength of children and was demonstrated for children aged 7–9 years within the 5th to 50th growth percentiles. By benchmarking growth trajectories against the IAP 2015 data and integrating nutrient intake ranges, the present validated model was able to simulate the effects of dietary interventions that target this at-risk population segment.

A key observation of the study is the variability in response to the intervention among different BMI types, emphasizing the importance of initial nutritional status in determining the effectiveness of interventions. Children classified as BMI Type 3 had the lowest baseline nutrient intake, meeting only ~35% of the RDA for all eight micronutrients, and exhibited the greatest improvements in growth outcomes. Their severe deficiencies initially resulted in energy allocation toward essential physiological functions rather than growth. As nutrient intake improved, energy was progressively redirected toward bone growth and muscle development, facilitating significant catch-up growth. This subgroup, characterized by low height and weight for age, benefited significantly from the MNB-M intervention, which provided 584 kcal, compared to the MNB-W intervention, which provided 155 kcal. Furthermore, out of the 8 micronutrients, iron contributed the most in height and weight improvement (~38%), followed by equal contributions from protein, and zinc (~25%). However, a minor penalty on height persisted in the BMI type 3 individuals of the MNB-M group as the %RDA of iodine intake was around 72% post-intervention, making ~12% contribution from iodine in the observed effects. This indicates scope for additional height gain in BMI type 3 population.

In contrast, BMI type 2 exhibited less severe deficiencies than the type 3 children at the baseline. BMI type 2 children were characterized by low height-for-age who exhibited deficiencies of iron and zinc, along with other functional nutrients as noted earlier. This BMI type responded well to the MNB-W as well as the MNB-M intervention. Specifically, the post-intervention improvements in height observed in this age group was majorly contributed by iron (~50%), followed by equal contributions from protein and zinc (~25%). Previously published trials examining the effects of intervention with these three micronutrients have shown height and weight improvements that closely align with the increments predicted by the model [17,37,38]. Additionally, ~ 28% contribution each from vitamin A, zinc, and protein, combined with a 14% contribution from folic acid, led to significant improvements in the lean mass-to-fat mass ratio.

BMI type 1 exhibited a more pronounced imbalance in their lean mass to fat mass ratio, which was more significant than the compromise observed in their height and weight growth. This was due to mild deficiencies in zinc and other functional nutrients at the baseline. Hence, this BMI type responded well to the MNB-W intervention, suggesting that even a lower-calorie intervention can bring in significant improvement from baseline for children with milder deficiencies. The lean mass improvement observed in the BMI Type 1 population was primarily driven by zinc (~50%), with protein contributing 33% and folic acid accounting for 16% of the total effect. Moreover, improvement in BMC is solely attributed to improved calcium intake compared to baseline.

The model predictions closely aligned with empirical data from previous studies. For instance, the outcomes observed in the study by Thomas et al. (2020), where children in the 25th to 50th BMI percentile received a multi-micronutrient intervention, showed an increase in height by 6.12 cm and weight by 3.6 kg over 12 months [39]. These results closely match the model’s predictions for the BMI type 1 population in the current study, which estimated a height gain of 6.36 cm and a weight gain of 4.2 kg under similar intervention conditions. Similarly, the study by Mehndiratta et al. (2021) demonstrated the efficacy of micronutrient-fortified milk in improving growth percentiles among children initially below the 25th BMI percentile [40]. The observed height velocity of 0.56 cm/month and weight gain of 400 g/month corresponded well with our model’s predictions for children classified as BMI type 3 receiving the MNB-M intervention, which estimated height and weight velocities of 0.53 cm/month and 358 g/month, respectively, under a similar 12-month intervention providing 584 kcal. These findings suggest that the model has effectively simulated the impact of nutrient interventions in populations with varying baseline growth statuses.

A notable strength of our *in-silico* approach is its ability to link macronutrient and micronutrient intake with specific anthropometric, body composition and strength parameters. The model’s capability to simulate the effect of MNB mix intervention and predict their impact on physical strength, as demonstrated by increases in HGS and standing jump performance, further underscores its utility in optimizing nutritional strategies. However, since the model is benchmarked against a population dataset of apparently healthy Indian children, it does not directly account for factors such as gut microbiome composition, socioeconomic status, healthcare access, and changes in baseline diet or dietary habits, all of which are known to influence child growth. As a result, these factors may contribute to variability in the results. Future research can benchmark and validate these factors to enhance the model’s robustness and improve its applicability across diverse populations. Additionally, while the model incorporates data from Indian children, generalization to other populations may be limited without further validation. Future studies could benefit from refining these assumptions and expanding the model’s application to diverse population groups. Despite its limitations, this *in-silico* study offers a valuable tool for predicting the effects of nutrient interventions on child growth. Notably, it is the first study to examine these effects based on varying anthropometric profiles within a detailed, simulation-based framework. The model’s strong alignment with empirical data further supports its potential application in designing effective nutritional interventions for children, particularly those at risk of undernutrition.

## Conclusions

This study underscores the value of model-based approaches in accurately predicting the effects of nutrient interventions on child growth, particularly in children across different BMI categories. The analysis of various BMI types highlighted the critical role of initial nutritional status in determining the effectiveness of intervention. Although both the MNB-W and MNB-M interventions proved beneficial by the end of the study, the MNB-M intervention, with its relatively higher energy and nutrient content, was better suited to address the more severe deficiencies observed in BMI type 3. Conversely, both MNB-W as well as the MNB-M intervention addressed milder deficiencies, particularly in children classified as BMI types 1 and 2. These findings underscore the need for targeted nutritional interventions to improve growth outcomes in children with nutritional deficiencies. Furthermore, they reinforce the importance of such strategies in public health initiatives aimed at addressing malnutrition and optimizing child health.

## Supporting information

S1 Table Baseline anthropometric and body composition parameters across all BMI types. (DOCX)

S2 Table Post-intervention (mean ± SD) anthropometric and body composition values. (DOCX)

S3 Table Between-group statistical comparison of biochemical outcomes across study timepoints. (DOCX)

S4 Table Post intervention (mean ± SD) anthropometric and body composition values as per growth percentiles. (DOCX)

S1 Fig Child growth model representation. (TIFF)

S2 Fig Validation of anthropometric and body composition parameters against clinical data. (a) Predicted height; (b) Predicted weight; (c) Body fat percentage; (d) BMC (bone mineral content). Straight line: 45o line; Dotted line: Error percentage.
(TIFF)

S3 Fig Scatterplot illustrating correlation between Fat-Free Mass (FFM) and Handgrip Strength (HGS) in the *in-silico* cohort. (TIFF)

S4 Fig Heatmap comparing degree of the penalty imposed by the nutrient deficiencies as per BMI types. (TIFF)

S5 Fig Validation of the biochemical platform using published clinical trials. (TIFF)

S6 Fig Post-intervention changes in biochemical parameters (w.r.t. baseline) among MNB-W group and MNB-M group. (TIFF)

S7 Fig Post-intervention changes in anthropometric changes among control group and MNB-M group. (TIFF)

S8 Fig Post-intervention changes in height velocity among control group and MNB-W group, stratified by growth percentiles. (TIFF)

S9 Fig Post-intervention changes in weight velocity among control group and MNB-W group, stratified by growth percentiles. (TIFF)

S10 Fig Post-intervention changes in delta lean mass to fat mass ratio among control group and MNB-W group, stratified by growth percentiles. (TIFF)

S11 Fig Post-intervention changes in bone mineral content (BMC) among control group and MNB-W group, stratified by growth percentiles. (TIFF)

S12 Fig Post-intervention changes in height velocity among MNB-WC group and MNB-W group, stratified by growth percentiles. (TIFF)

S13 Fig Post-intervention changes in weight velocity among MNB-WC group and MNB-W group, stratified by growth percentiles. (TIFF)

S14 Fig Post-intervention changes in delta lean mass to fat mass ratio among MNB-WC group and MNB-W group, stratified by growth percentiles. (TIFF)

S15 Fig Post-intervention changes in bone mineral content (BMC) among MNB-WC group and MNB-W group, stratified by growth percentiles. (TIFF)

S16 Fig Post-intervention changes in height velocity among control group and MNB-M group, stratified by growth percentiles. (TIFF)

S17 Fig Post-intervention changes in weight velocity among control group and MNB-M group, stratified by growth percentiles. (TIFF)

S18 Fig Post-intervention changes in delta lean mass to fat mass ratio among control group and MNB-M group, stratified by growth percentiles. (TIFF)

S19 Fig Post-intervention changes in change in change in bone mineral content (BMC) among control group and MNB-M group, stratified by growth percentiles. (TIFF)
