# Supplementary material for: Evaluation of a micro-nutrient beverage mix intervention on biochemical parameters, growth, and strength in Indian children with diverse anthropometric profiles: An in-silico study
Source: PLoS One. 2025 Aug 25;20(8):e0318629. doi: 10.1371/journal.pone.0318629 (PMC12377616; doi:10.1371/journal.pone.0318629)
Supplement: S1 Table — (DOCX) [file pone.0318629.s001.docx]

**SUPPLEMENTARY TABLE**

Table S1: Baseline anthropometric and body composition parameters across all BMI types.

| **Outcome Parameters** | **Baseline** | |
| --- | --- | --- |
| Growth percentiles | 5^th^ to 50^th^ | |
| Height (cm) | BMI Type 1 | 120.0 ± 6.2 |
|  | BMI Type 2 | 115.2 ± 4.2 |
|  | BMI Type 3 | 116.1 ± 5.5 |
| Weight (kg) | BMI Type 1 | 21.6 ± 2.6 |
|  | BMI Type 2 | 22.0 ± 2.4 |
|  | BMI Type 3 | 18.4 ± 1.9 |
| BMI (kg/m^2^) | BMI Type 1 | 15.0 ± 0.4 |
|  | BMI Type 2 | 16.5 ± 0.8 |
|  | BMI Type 3 | 13.6 ± 0.2 |
| Lean mass (kg) | BMI Type 1 | 17.3 ± 2.1 |
|  | BMI Type 2 | 15.9 ± 1.6 |
|  | BMI Type 3 | 15.8 ± 1.6 |
| Fat mass (kg) | BMI Type 1 | 3.5 ± 0.9 |
|  | BMI Type 2 | 5.3 ± 1.2 |
|  | BMI Type 3 | 1.9 ± 0.5 |
| Bone mineral content (g) | BMI Type 1 | 784.9 ± 138.9 |
|  | BMI Type 2 | 730.5 ± 107.8 |
|  | BMI Type 3 | 644.3 ± 65.9 |
| Hand grip strength (kg) | BMI Type 1 | 10.4 ± 1.4 |
|  | BMI Type 2 | 9.6 ± 1.1 |
|  | BMI Type 3 | 9.5 ± 1.1 |
| Standing long jump (cm.) | BMI Type 1 | 126.1 ± 5.2 |
|  | BMI Type 2 | 123.6 ± 4.6 |
|  | BMI Type 3 | 123.2 ± 4.3 |
