# Supplementary material for: Evaluation of a micro-nutrient beverage mix intervention on biochemical parameters, growth, and strength in Indian children with diverse anthropometric profiles: An in-silico study
Source: PLoS One. 2025 Aug 25;20(8):e0318629. doi: 10.1371/journal.pone.0318629 (PMC12377616; doi:10.1371/journal.pone.0318629)
Supplement: S2 Table — (DOCX) [file pone.0318629.s002.docx]

**SUPPLEMENTARY TABLE**

Table S2: Post-intervention (mean ± SD) anthropometric and body composition values:

| **Parameters** | **BMI Type 1** | | | | **BMI Type 2** | | | | **BMI Type 3** | | | |
| --- | --- | --- | --- | --- | --- | --- | --- | --- | --- | --- | --- | --- |
|  | **Control** | **2 PRDT** | **2 PRDT (CPF)** | **2 PRDT 2 MILK** | **Control** | **2 PRDT** | **2 PRDT (CPF)** | **2 PRDT 2 MILK** | **Control** | **2 PRDT** | **2 PRDT (CPF)** | **2 PRDT 2 MILK** |
| Height (cm) | 124.3 ± 6.5 | 124.4 ± 6.5 | 124.3 ± 6.5 | 126.4 ± 5.6 | 119.1 ± 4.0 | 119.3 ± 4.1 | 119.1 ± 4.0 | 121.5 ± 3.8 | 120.0 ± 5.4 | 120.0 ± 5.4 | 120.0 ± 5.4 | 123.3 ± 5.1 |
| Weight (kg) | 22.9 ± 2.9 | 23.6 ± 2.8 | 23.5 ± 2.8 | 25.9 ± 2.6 | 23.3 ± 2.7 | 24.1 ± 2.6 | 23.9 ± 2.6 | 26.1 ± 2.6 | 19.1 ± 2.0 | 19.8 ± 2.0 | 19.1 ± 1.9 | 22.8 ± 2.0 |
| BMI (kg/m2) | 14.8 ± 0.5 | 15.2 ± 0.5 | 15.2 ± 0.5 | 16.1 ± 0.6 | 16.3 ± 0.9 | 16.8 ± 0.8 | 16.8 ± 0.8 | 17.7 ± 0.8 | 13.2 ± 0.3 | 13.7 ± 0.2 | 13.2 ± 0.3 | 15.0 ± 0.2 |
| Fat mass (kg) | 3.9 ± 1.1 | 4.0 ± 1.1 | 4.0 ± 1.1 | 4.2 ± 1.2 | 5.7 ± 1.3 | 5.8 ± 1.4 | 5.9 ± 1.4 | 6.0 ± 1.4 | 2.1 ± 0.6 | 2.1 ± 0.6 | 2.0 ± 0.6 | 2.4 ± 0.8 |
| Bone mineral content (g) | 831.3 ± 153.4 | 901.4 ± 120.4 | 852.8 ± 151.1 | 1008.9 ± 103.3 | 773.5 ± 118.4 | 916.5 ± 112.1 | 1019.1 ± 101.7 | 794.4 ± 115.7 | 669.3 ± 68.7 | 773.5 ± 77.2 | 669.0 ± 68.4 | 889.8 ± 79.6 |
| Lean mass (kg) | 18.2 ± 2.3 | 18.8 ± 2.2 | 18.6 ± 2.2 | 20.7 ± 2.1 | 16.8 ± 1.8 | 17.3 ± 1.8 | 17.2 ± 1.8 | 19.1 ± 1.9 | 16.3 ± 1.6 | 16.9 ± 1.6 | 16.3 ± 1.6 | 19.6 ± 1.7 |
| Hand grip strength (kg) | 11 ± 1.5 | 11.3 ± 1.5 | 11.3 ± 1.5 | 12.4 ± 1.5 | 10.1 ± 1.2 | 10.5 ± 1.2 | 10.4 ± 1.2 | 11.6 ± 1.3 | 9.5 ± 1.1 | 9.7 ± 1.1 | 9.5 ± 1.1 | 10.3 ± 1.2 |
| Standing long jump (cm.) | 125.1 ± 4.8 | 126.3 ± 4.9 | 126.1 ± 4.9 | 129.6 ± 5.2 | 127.6 ± 5.5 | 128.7 ± 5.6 | 128.6 ± 5.6 | 132.2 ± 5.7 | 124.1 ± 4.3 | 125.3 ± 4.5 | 124.2 ± 4.3 | 130.1 ± 5 |
