# Supplementary material for: Evaluation of a micro-nutrient beverage mix intervention on biochemical parameters, growth, and strength in Indian children with diverse anthropometric profiles: An in-silico study
Source: PLoS One. 2025 Aug 25;20(8):e0318629. doi: 10.1371/journal.pone.0318629 (PMC12377616; doi:10.1371/journal.pone.0318629)
Supplement: S3 Table — (DOCX) [file pone.0318629.s003.docx]

**SUPPLEMENTARY TABLE**

Table S3: Between-Group Statistical Comparison of Biochemical Outcomes Across Study Timepoints.

|  | **4 months** | | | **8 months** | | | **12 months** | | |
| --- | --- | --- | --- | --- | --- | --- | --- | --- | --- |
|  | **MNB-M vs MNB-W** | | | | | | | | |
|  | **t-stats** | **p-value** | **Cohen's D** | **t-stats** | **p-value** | **Cohen's D** | **t-stats** | **p-value** | **Cohen's D** |
| Serum Calcium | 5 | < 0.0001 | 0.2 | 10 | < 0.0001 | 0.3 | 14 | < 0.0001 | 0.5 |
| Serum Ferritin | 8 | < 0.0001 | 0.2 | 16 | < 0.0001 | 0.5 | 23 | < 0.0001 | 0.7* |
| Urinary Iodine | 0 | 1 | 0 | 0 | 1 | 0 | 0 | 1 | 0 |
| Serum Selenium | 11 | < 0.0001 | 0.3 | 21 | < 0.0001 | 0.7* | 31 | < 0.0001 | 1* |
| Serum Vitamin A | 75 | < 0.0001 | 2.4* | 153 | < 0.0001 | 4.8* | 225 | < 0.0001 | 7.1* |
| Serum Vitamin B9 | 6 | < 0.0001 | 0.2 | 12 | < 0.0001 | 0.4 | 16 | < 0.0001 | 0.5 |
| Serum Vitamin D | 65 | < 0.0001 | 2* | 149 | < 0.0001 | 4.7* | 181 | < 0.0001 | 5.7* |
| Serum Zinc | 169 | < 0.0001 | 5.4* | 348 | < 0.0001 | 11* | 520 | < 0.0001 | 16.4* |

*Large effect size.
