# Supplementary material for: Evaluation of a micro-nutrient beverage mix intervention on biochemical parameters, growth, and strength in Indian children with diverse anthropometric profiles: An in-silico study
Source: PLoS One. 2025 Aug 25;20(8):e0318629. doi: 10.1371/journal.pone.0318629 (PMC12377616; doi:10.1371/journal.pone.0318629)
Supplement: S4 Table — (DOCX) [file pone.0318629.s004.docx]

**SUPPLEMENTARY TABLE**

Table S4: Post intervention (mean ± SD) anthropometric and body composition values as per growth percentiles.

| **Parameters** | **5th to 25th percentile** | | | | **25th to 50th percentile** | | | |
| --- | --- | --- | --- | --- | --- | --- | --- | --- |
|  | **Control** | **MNB-W** | **MNB-WC** | **MNB-M** | **Control** | **MNB-W** | **MNB-WC** | **MNB-M** |
|  | **BMI Type 1** | | | | | | | |
| Height (cm) | 120.1 ± 4.3 | 120.1 ± 4.3 | 120.0 ± 4.3 | 123.2 ± 4.1 | 128.6 ± 5.5 | 128.7 ± 5.5 | 128.6 ± 5.5 | 129.6 ± 5.1 |
| Weight (kg) | 21.2 ± 2.0 | 22.1 ± 2.1 | 22.0 ± 2.1 | 24.9 ± 2.2 | 24.6 ± 2.6 | 25.1 ± 2.6 | 25.0 ± 2.6 | 26.9 ± 2.7 |
| BMI (kg/m2) | 14.7 ± 0.5 | 15.3 ± 0.5 | 15.2 ± 0.5 | 16.4 ± 0.5 | 14.8 ± 0.6 | 15.1 ± 0.6 | 15.1 ± 0.6 | 15.9 ± 0.5 |
| Fat mass (kg) | 3.6 ± 0.9 | 3.7 ± 1.0 | 3.7 ± 1.0 | 3.9 ± 1.1 | 4.2 ± 1.1 | 4.3 ± 1.1 | 4.3 ± 1.1 | 4.4 ± 1.2 |
| Bone mineral content (g) | 704.4 ± 67.8 | 822.1 ± 79.5 | 729.0 ± 70.5 | 970.6 ± 86.9 | 958.3 ± 101.3 | 980.7 ± 100.4 | 976.6 ± 100.1 | 1047.2 ± 104.2 |
| Lean mass (kg) | 17.0 ± 1.7 | 17.6 ± 1.8 | 17.5 ± 1.8 | 20.0 ± 1.9 | 19.4 ± 2.1 | 19.9 ± 2.1 | 19.8 ± 2.1 | 21.4 ± 2.1 |
|  | **BMI Type 2** | | | | | | | |
| Height (cm) | 118.2 ± 3.6 | 118.3 ± 3.7 | 118.2 ± 3.6 | 121.2 ± 3.6 | 120.1 ± 4.2 | 120.4 ± 4.2 | 120.1 ± 4.2 | 121.7 ± 4.1 |
| Weight (kg) | 22.0 ± 2.0 | 22.9 ± 2.2 | 22.8 ± 2.2 | 25.4 ± 2.3 | 24.6 ± 2.6 | 25.2 ± 2.6 | 25.0 ± 2.6 | 26.9 ± 2.7 |
| BMI (kg/m2) | 15.7 ± 0.5 | 16.3 ± 0.6 | 16.2 ± 0.6 | 17.2 ± 0.6 | 17.0 ± 0.7 | 17.3 ± 0.7 | 17.3 ± 0.7 | 18.1 ± 0.7 |
| Fat mass (kg) | 5.0 ± 1.0 | 5.1 ± 1.0 | 5.2 ± 1.0 | 5.4 ± 1.1 | 6.4 ± 1.3 | 6.5 ± 1.3 | 6.5 ± 1.3 | 6.6 ± 1.4 |
| Bone mineral content (g) | 685.1 ± 63.7 | 851.3 ± 80.6 | 710.4 ± 67.3 | 989.6 ± 89.9 | 862.0 ± 91.3 | 981.8 ± 100.6 | 878.4 ± 90.1 | 1048.6 ± 104.3 |
| Lean mass (kg) | 16.3 ± 1.5 | 17.0 ± 1.6 | 16.8 ± 1.6 | 19.0 ± 1.8 | 17.3 ± 1.9 | 17.7 ± 1.9 | 17.6 ± 1.9 | 19.2 ± 1.9 |
|  | **BMI Type 3** | | | | | | | |
| Height (cm) | 120.0 ± 5.4 | 120.0 ± 5.4 | 120.0 ± 5.4 | 123.3 ± 5.1 | - | - | - | - |
| Weight (kg) | 19.1 ± 2.0 | 19.8 ± 2.0 | 19.1 ± 1.9 | 22.8 ± 2.0 | - | - | - | - |
| BMI (kg/m2) | 13.2 ± 0.3 | 13.7 ± 0.2 | 13.2 ± 0.3 | 15.0 ± 0.2 | - | - | - | - |
| Fat mass (kg) | 2.1 ± 0.6 | 2.1 ± 0.6 | 2.0 ± 0.6 | 2.4 ± 0.8 | - | - | - | - |
| Bone mineral content (g) | 669.3 ± 68.7 | 773.5 ± 77.2 | 669.0 ± 68.4 | 889.8 ± 79.6 | - | - | - | - |
| Lean mass (kg) | 16.3 ± 1.6 | 16.9 ± 1.6 | 16.3 ± 1.6 | 19.6 ± 1.7 | - | - | - | - |
