# Supplementary figures and images for: Evaluation of a micro-nutrient beverage mix intervention on biochemical parameters, growth, and strength in Indian children with diverse anthropometric profiles: An in-silico study
Source: PLoS One. 2025 Aug 25;20(8):e0318629. doi: 10.1371/journal.pone.0318629 (PMC12377616; doi:10.1371/journal.pone.0318629)

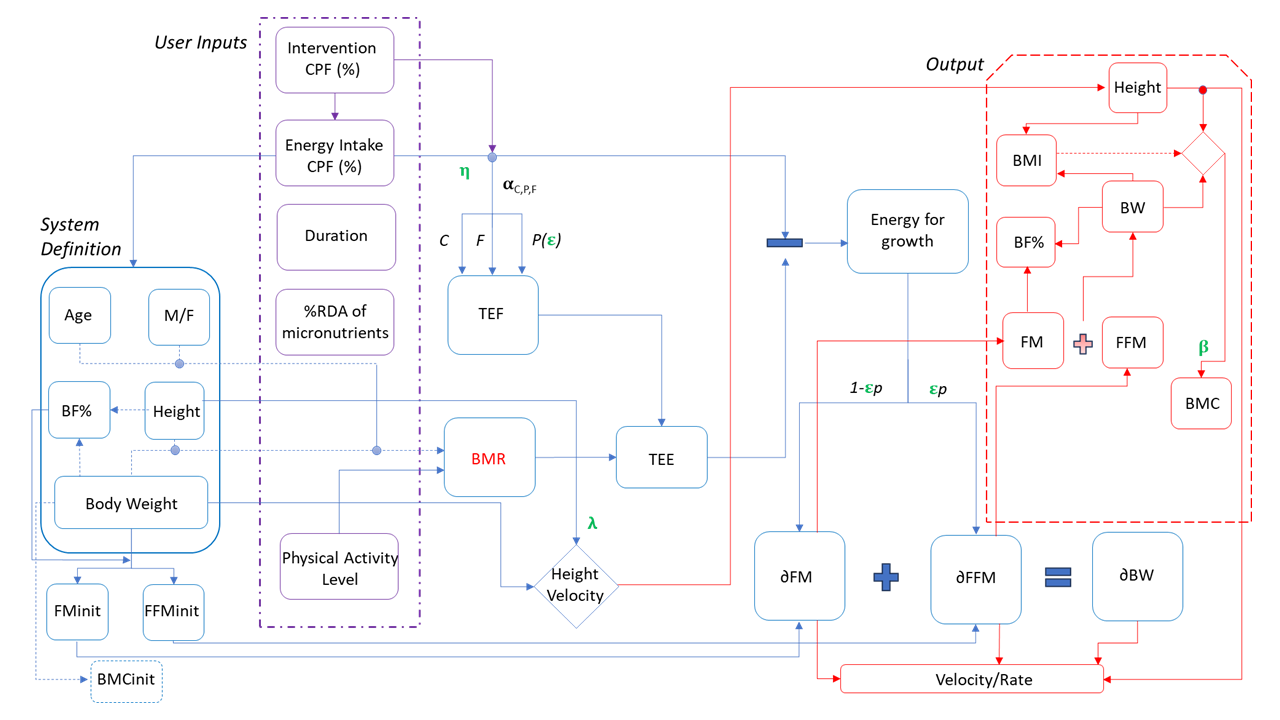

Supplement: S1 Fig — (TIFF) [file pone.0318629.s005.tiff]

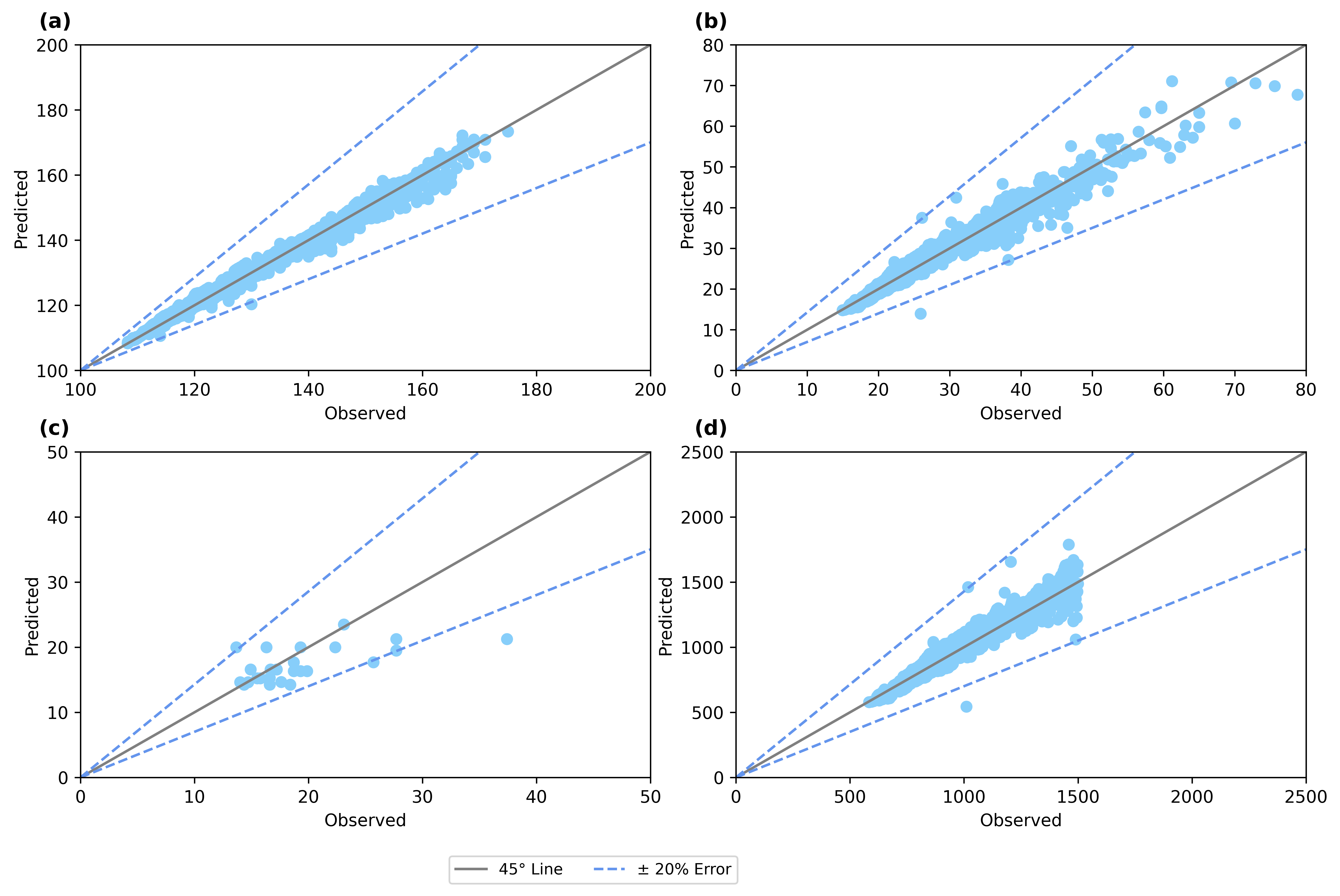

Supplement: S2 Fig — (a) Predicted height; (b) Predicted weight; (c) Body fat percentage; (d) BMC (bone mineral content). Straight line: 45o line; Dotted line: Error percentage. (TIFF) [file pone.0318629.s006.tiff]

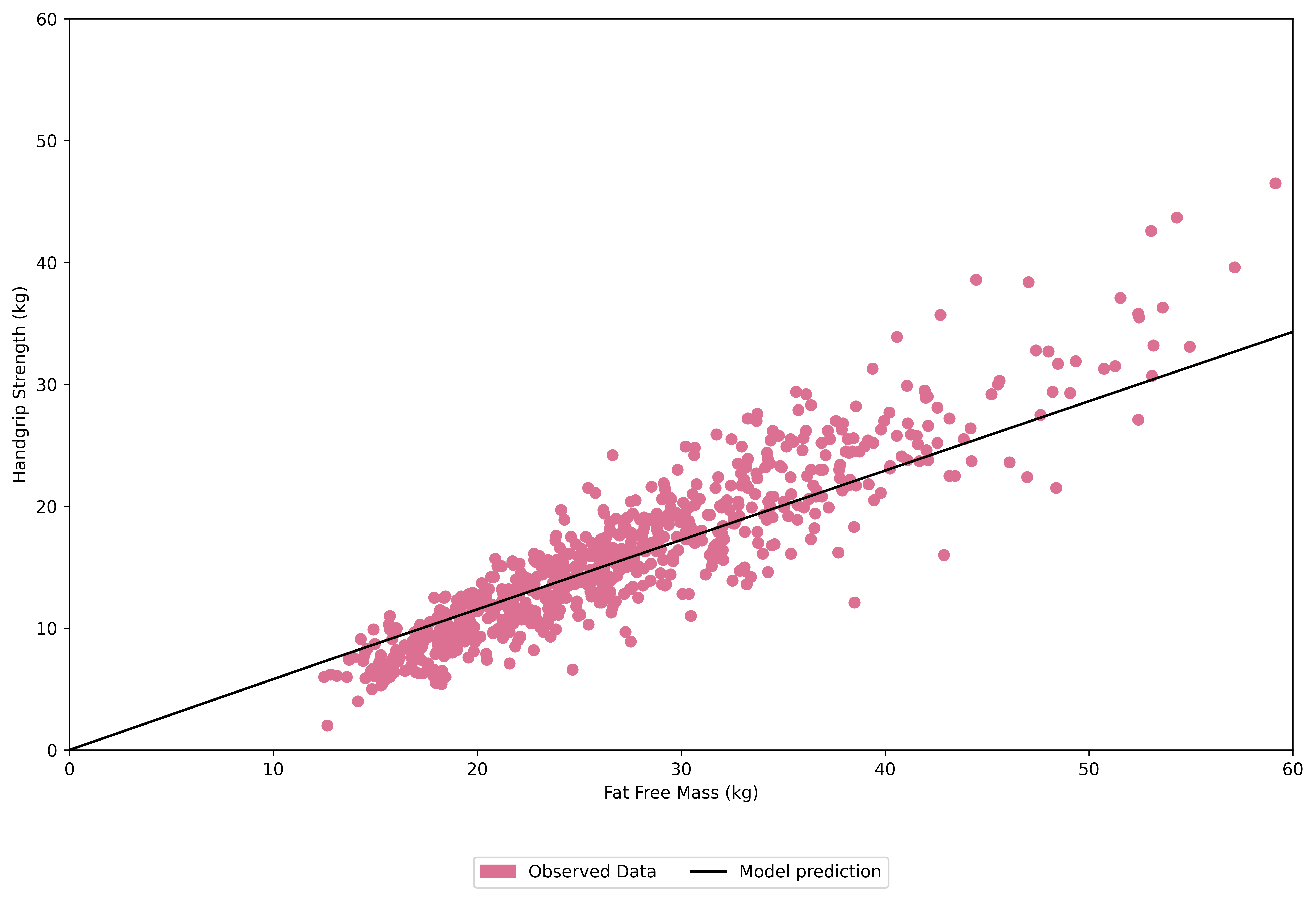

Supplement: S3 Fig — (TIFF) [file pone.0318629.s007.tiff]

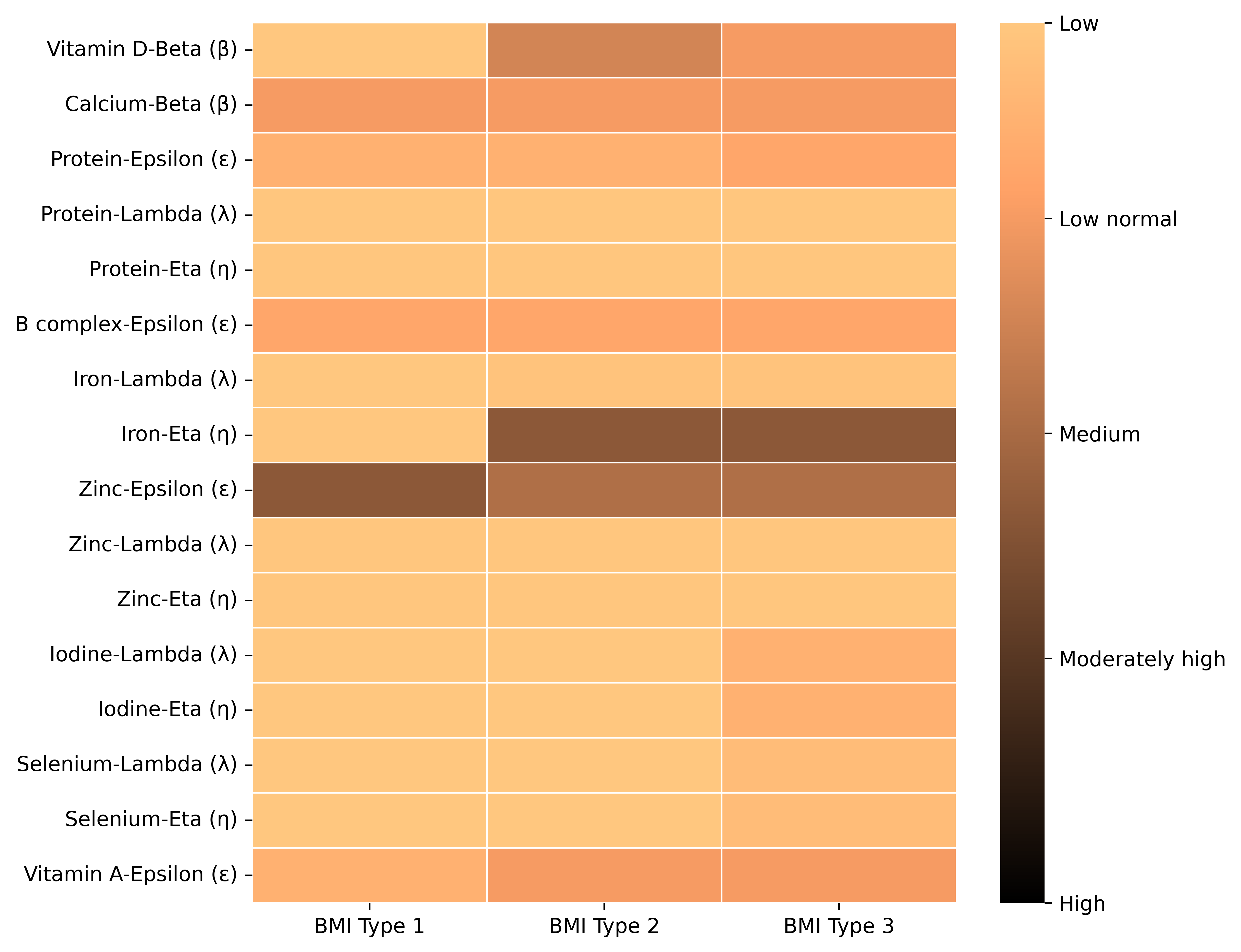

Supplement: S4 Fig — (TIFF) [file pone.0318629.s008.tiff]

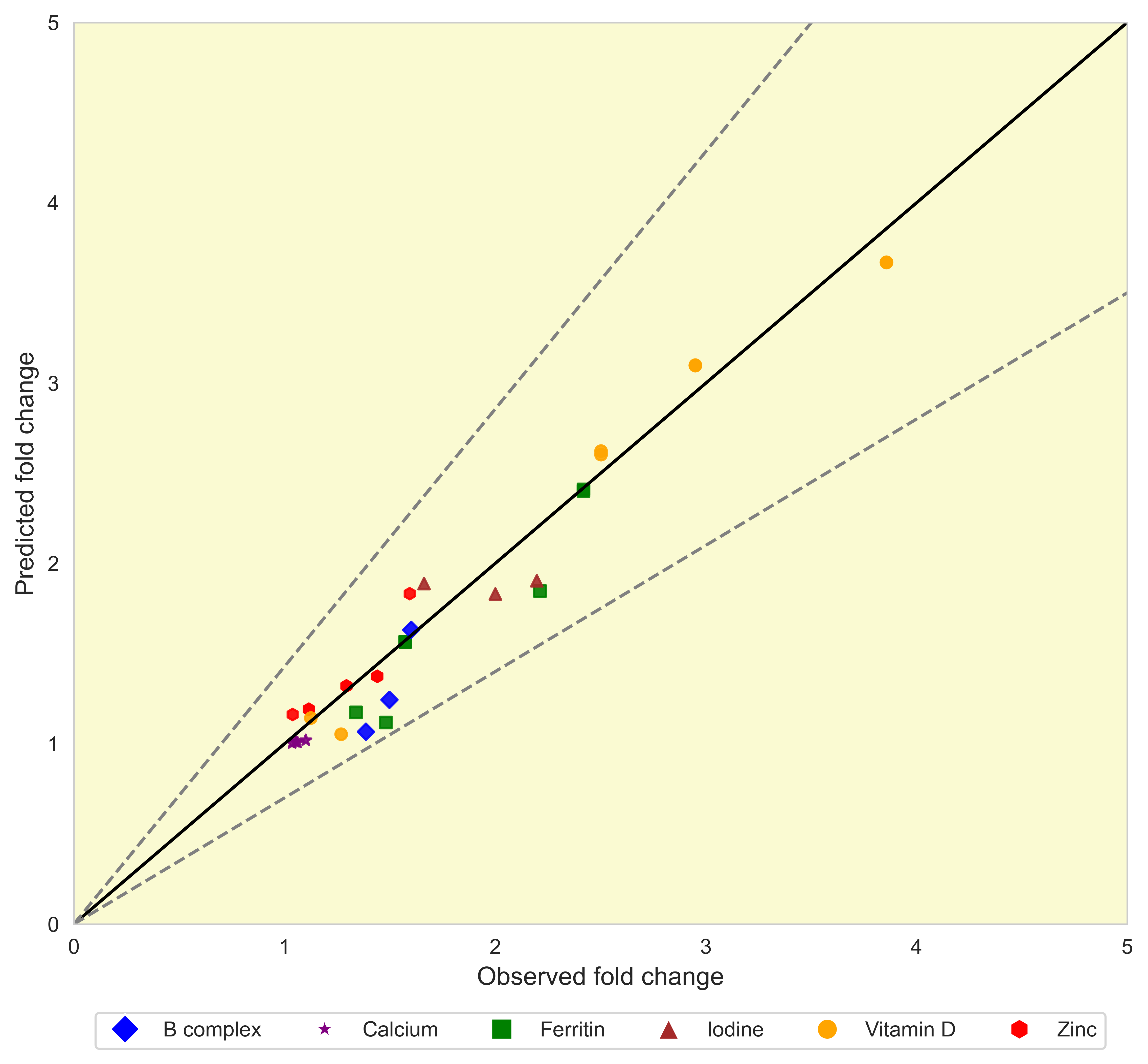

Supplement: S5 Fig — (TIFF) [file pone.0318629.s009.tiff]

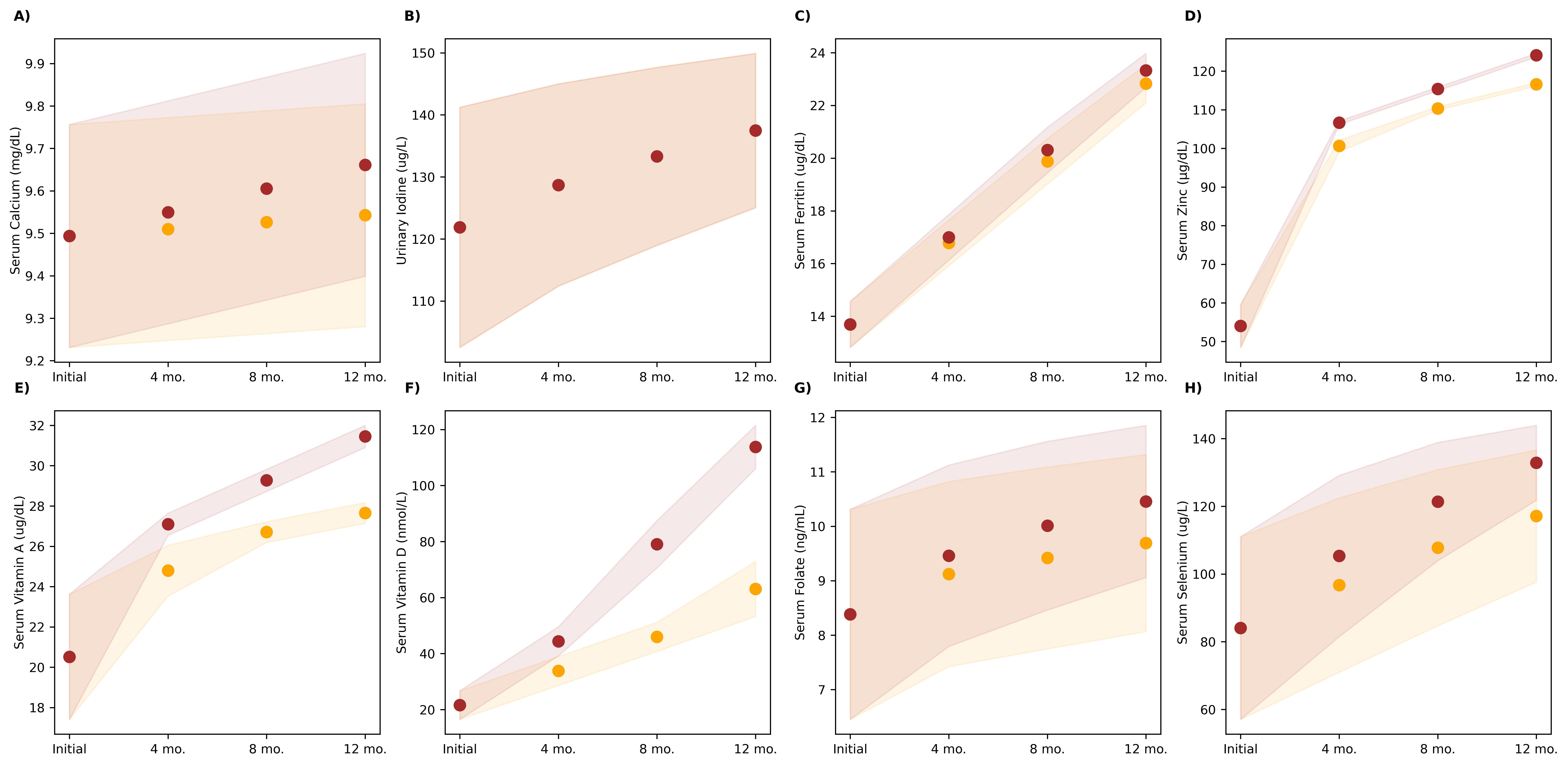

Supplement: S6 Fig — (TIFF) [file pone.0318629.s010.tiff]

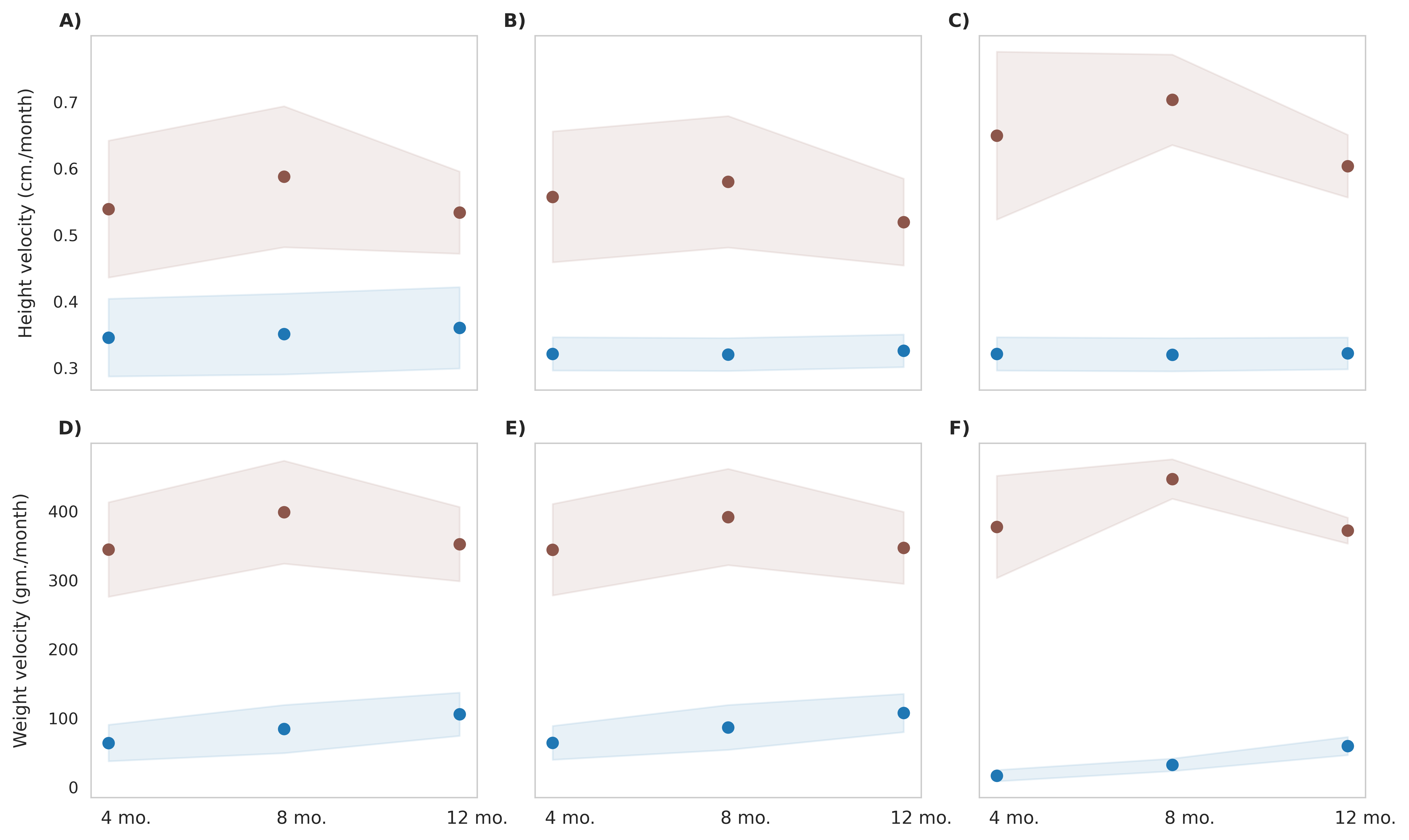

Supplement: S7 Fig — (TIFF) [file pone.0318629.s011.tiff]

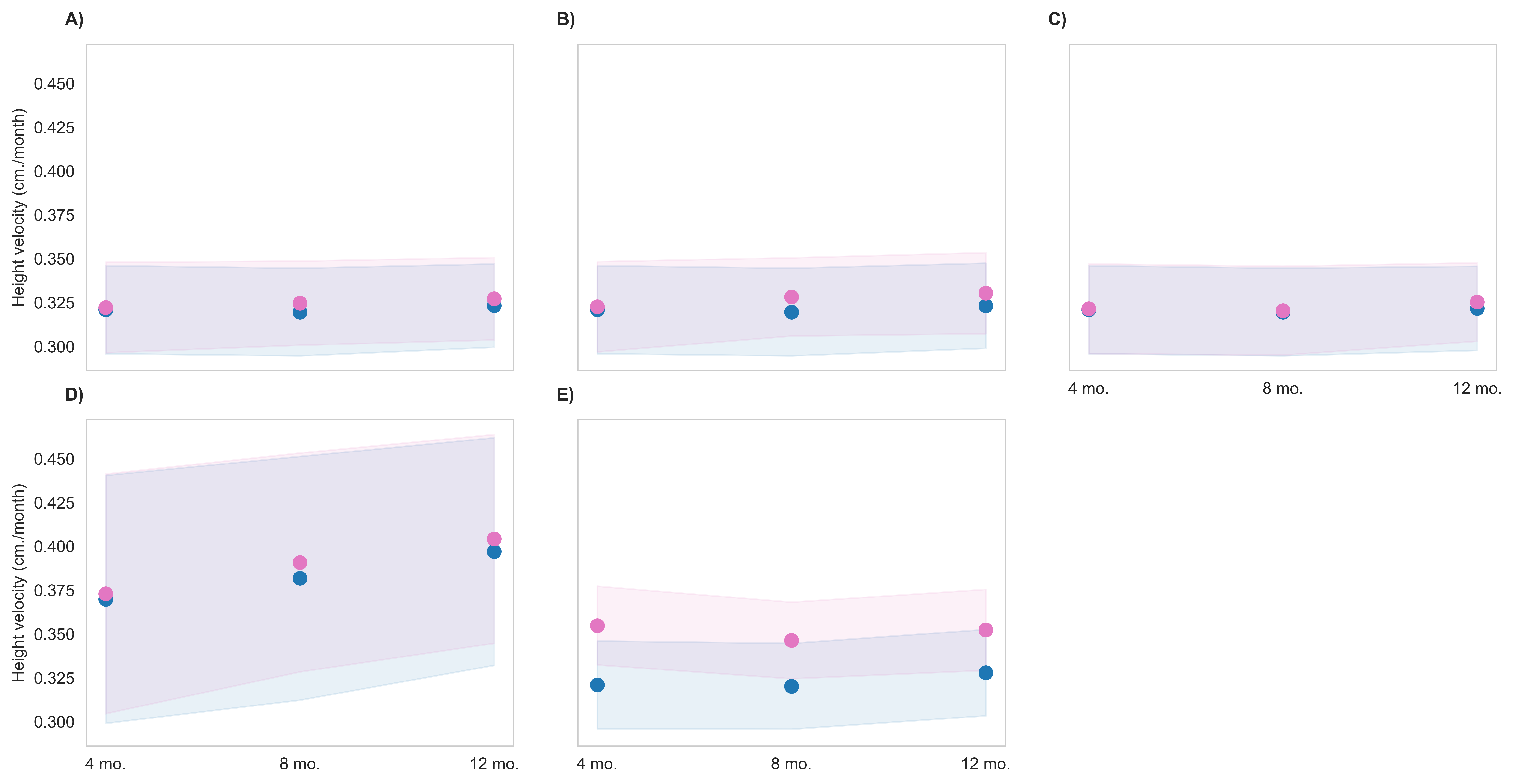

Supplement: S8 Fig — (TIFF) [file pone.0318629.s012.tiff]

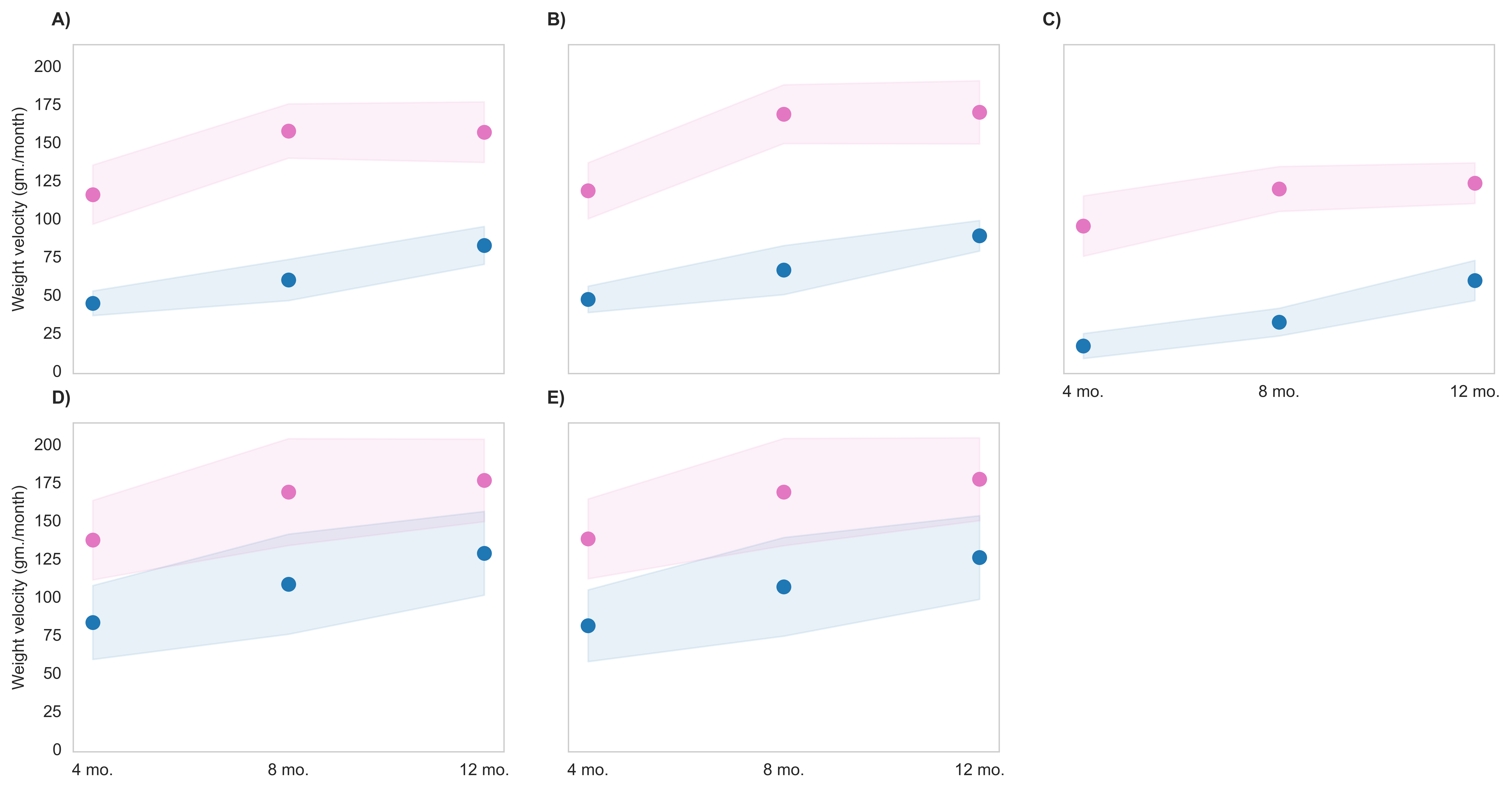

Supplement: S9 Fig — (TIFF) [file pone.0318629.s013.tiff]

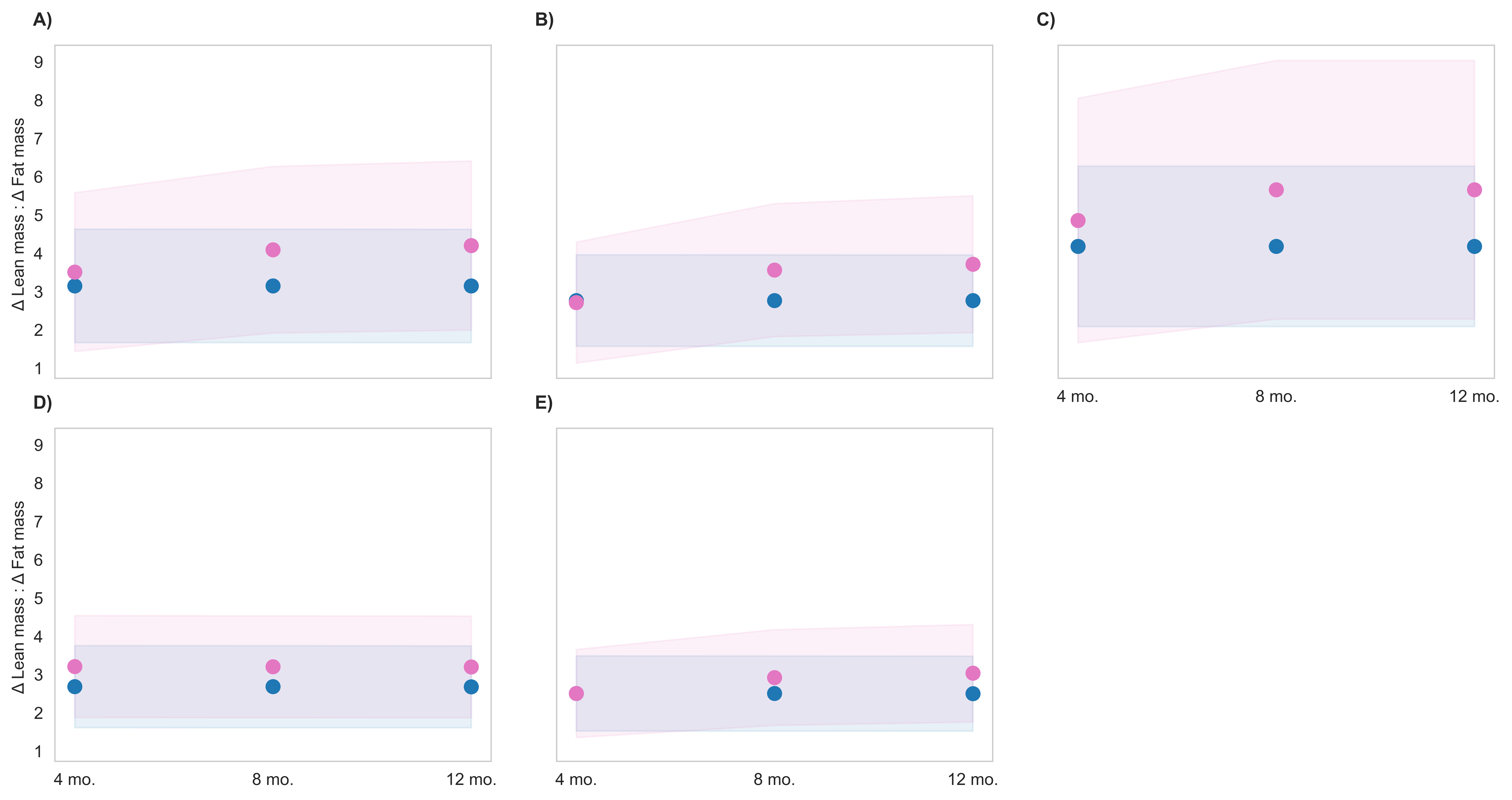

Supplement: S10 Fig — (TIFF) [file pone.0318629.s014.tiff]

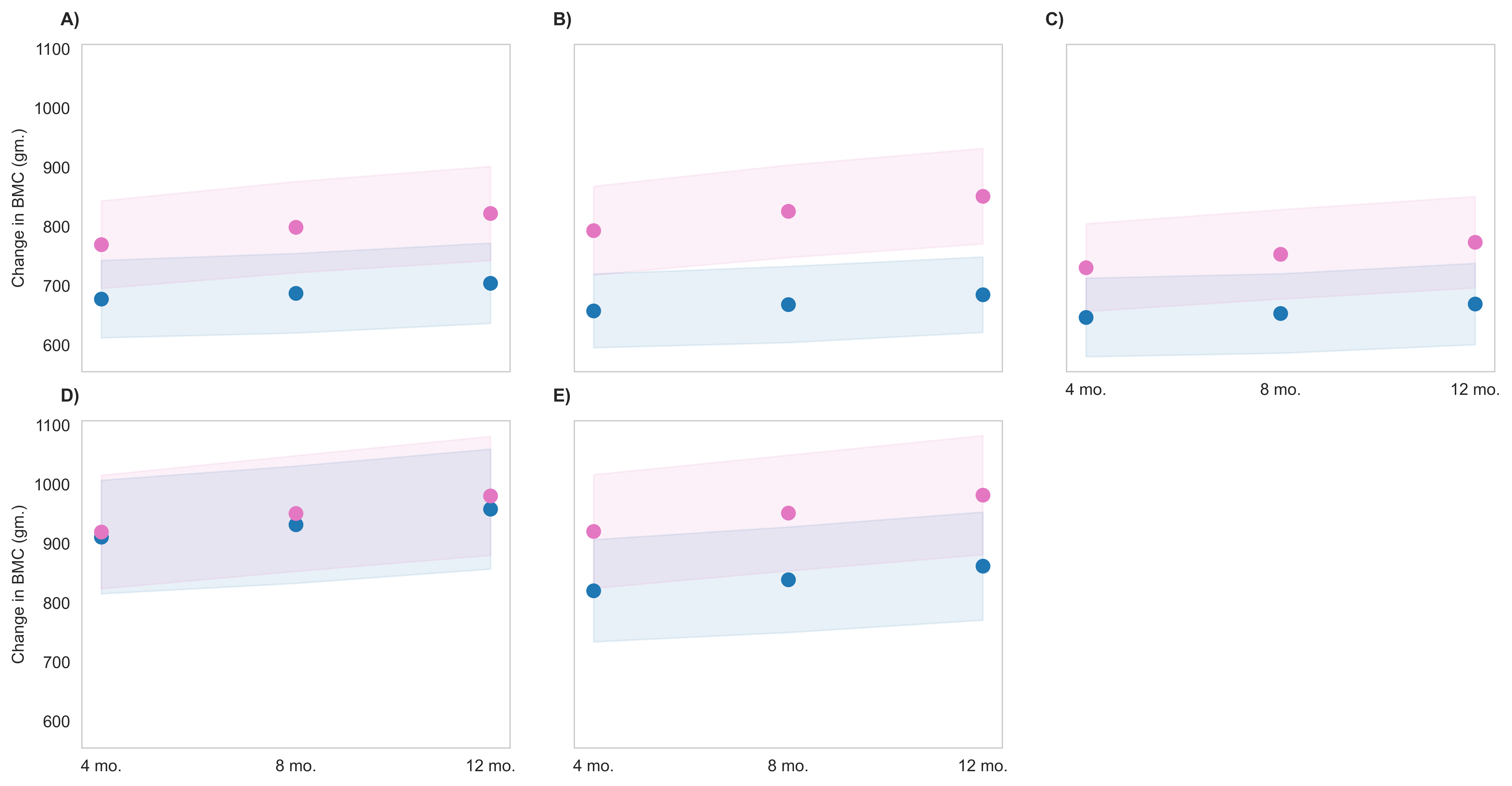

Supplement: S11 Fig — (TIFF) [file pone.0318629.s015.tiff]

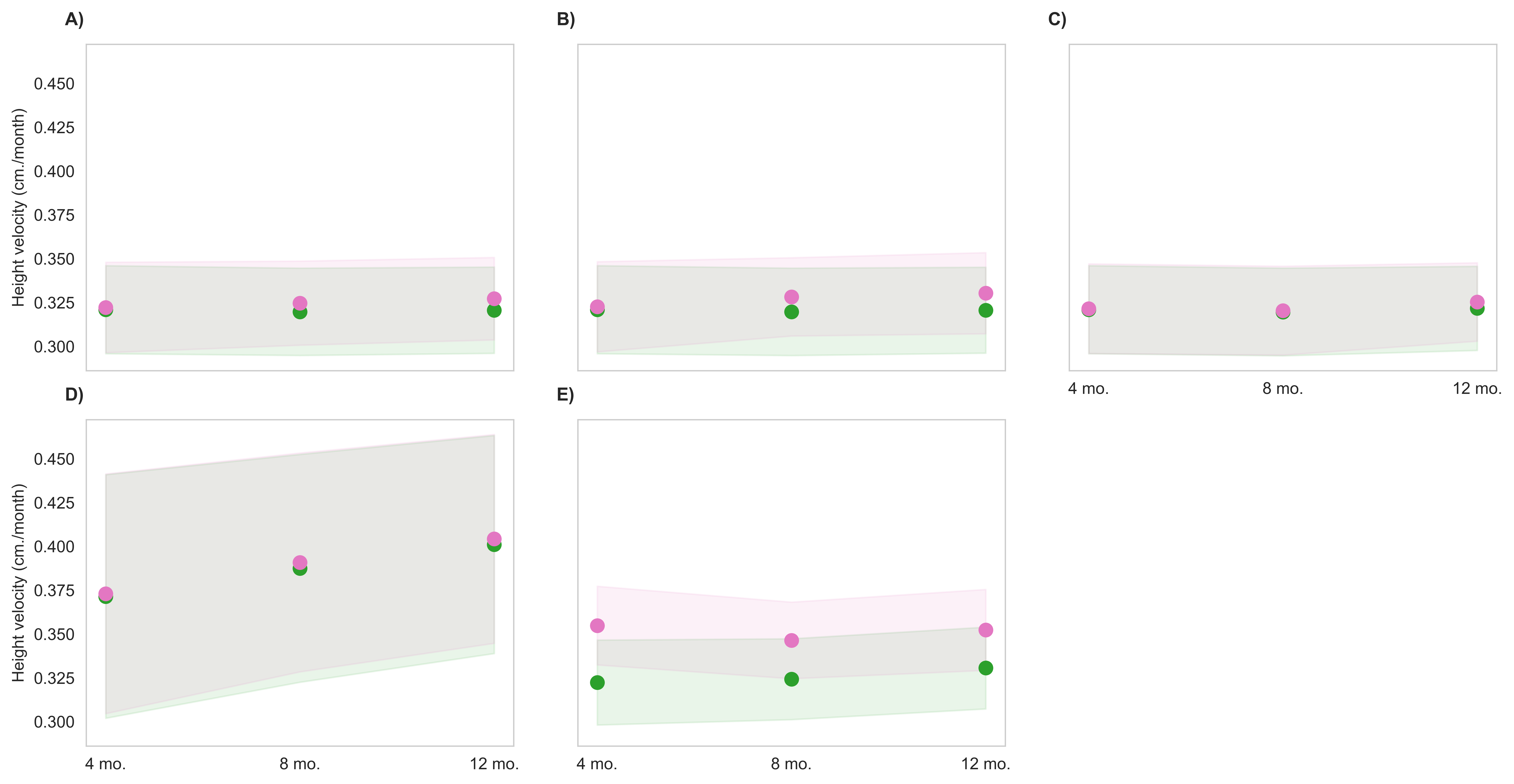

Supplement: S12 Fig — (TIFF) [file pone.0318629.s016.tiff]

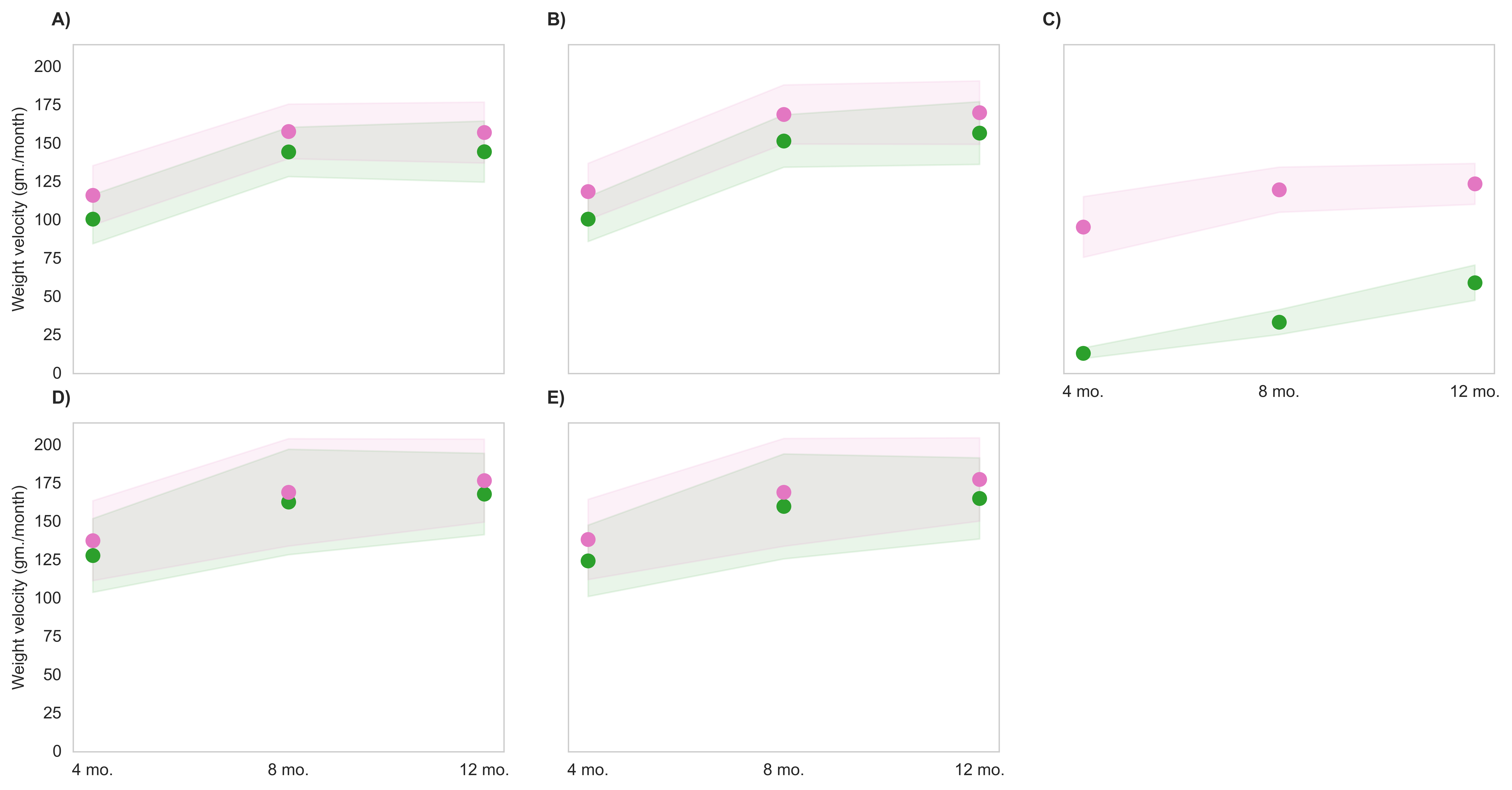

Supplement: S13 Fig — (TIFF) [file pone.0318629.s017.tiff]

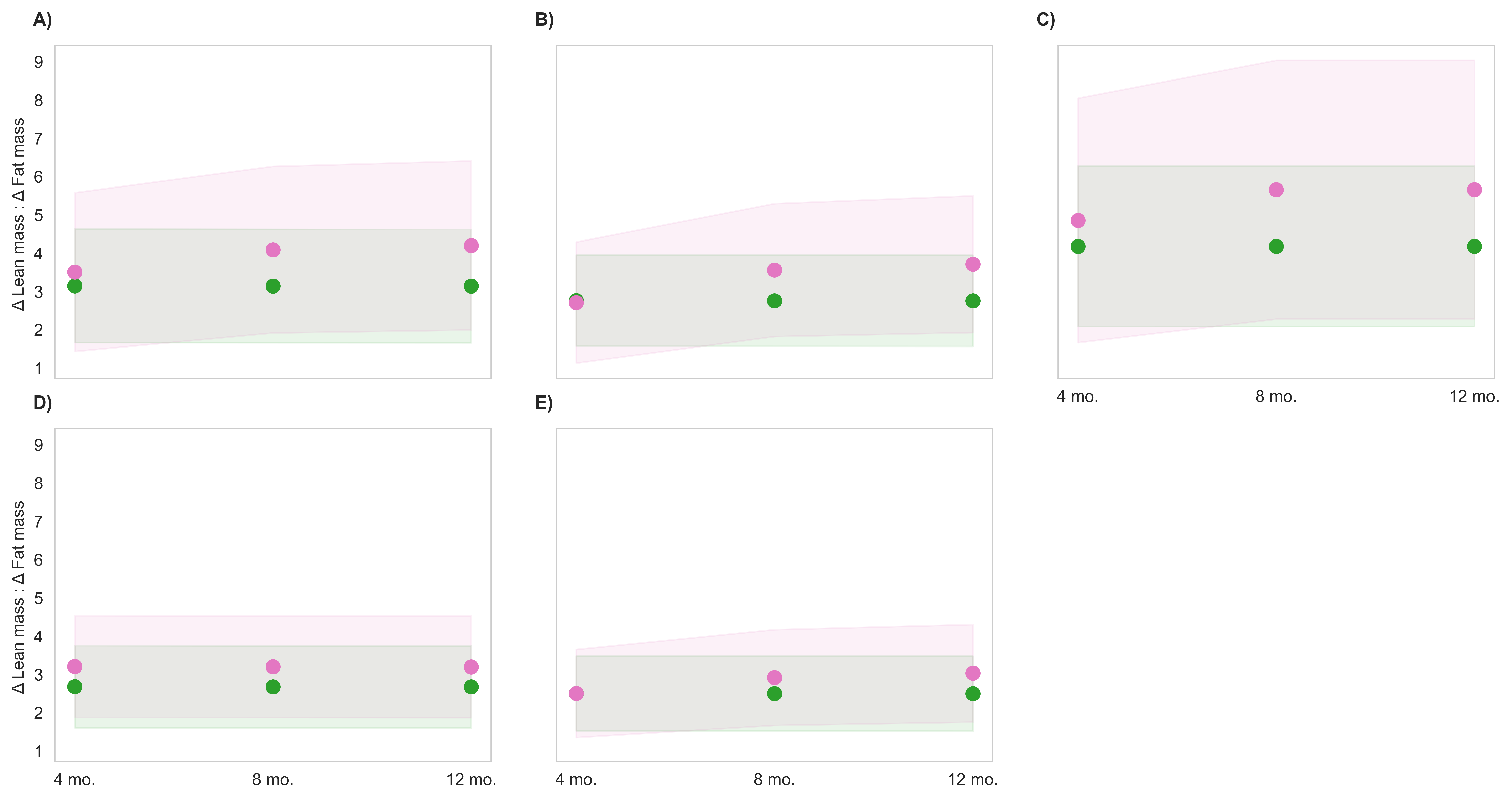

Supplement: S14 Fig — (TIFF) [file pone.0318629.s018.tiff]

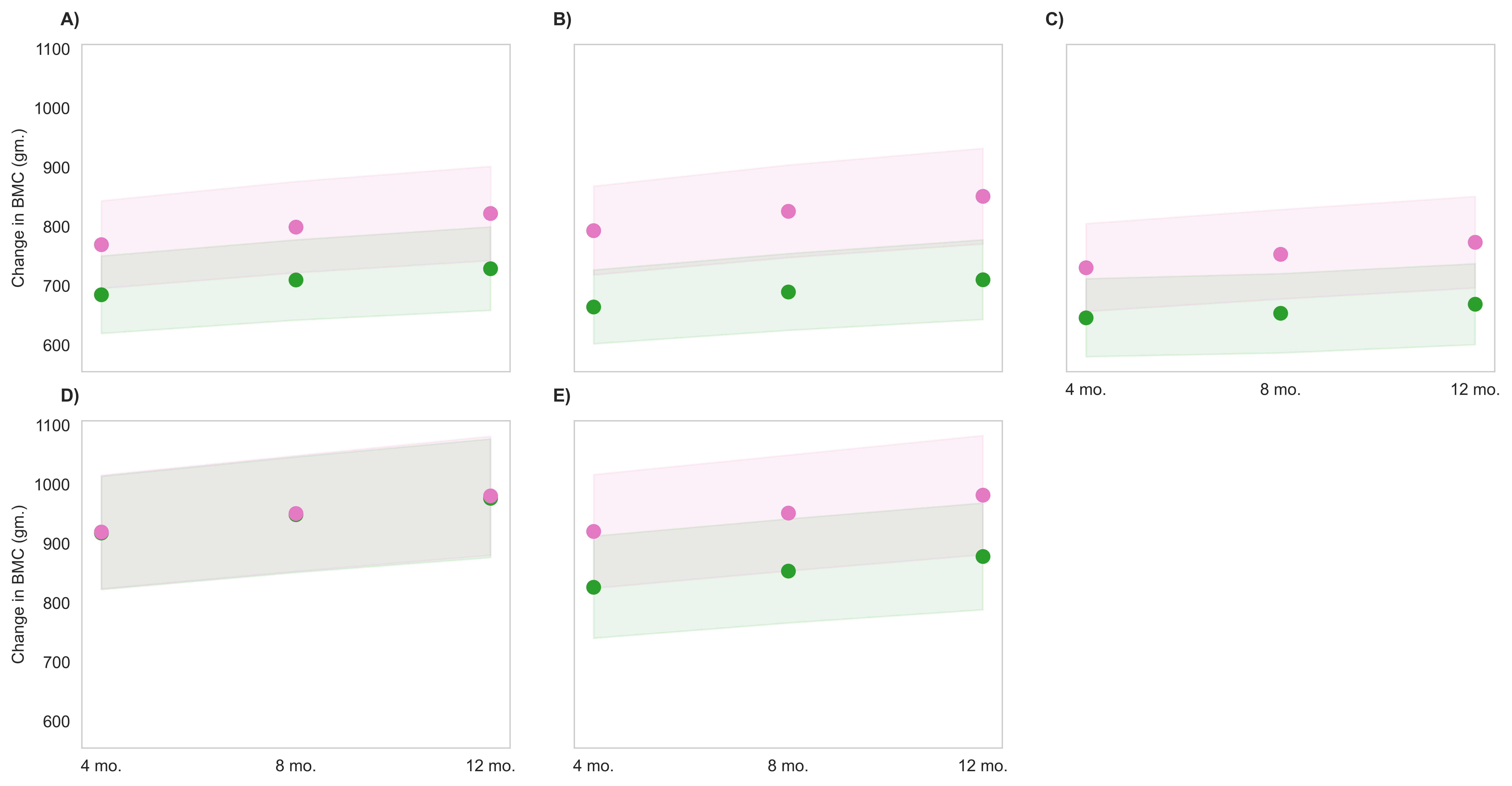

Supplement: S15 Fig — (TIFF) [file pone.0318629.s019.tiff]

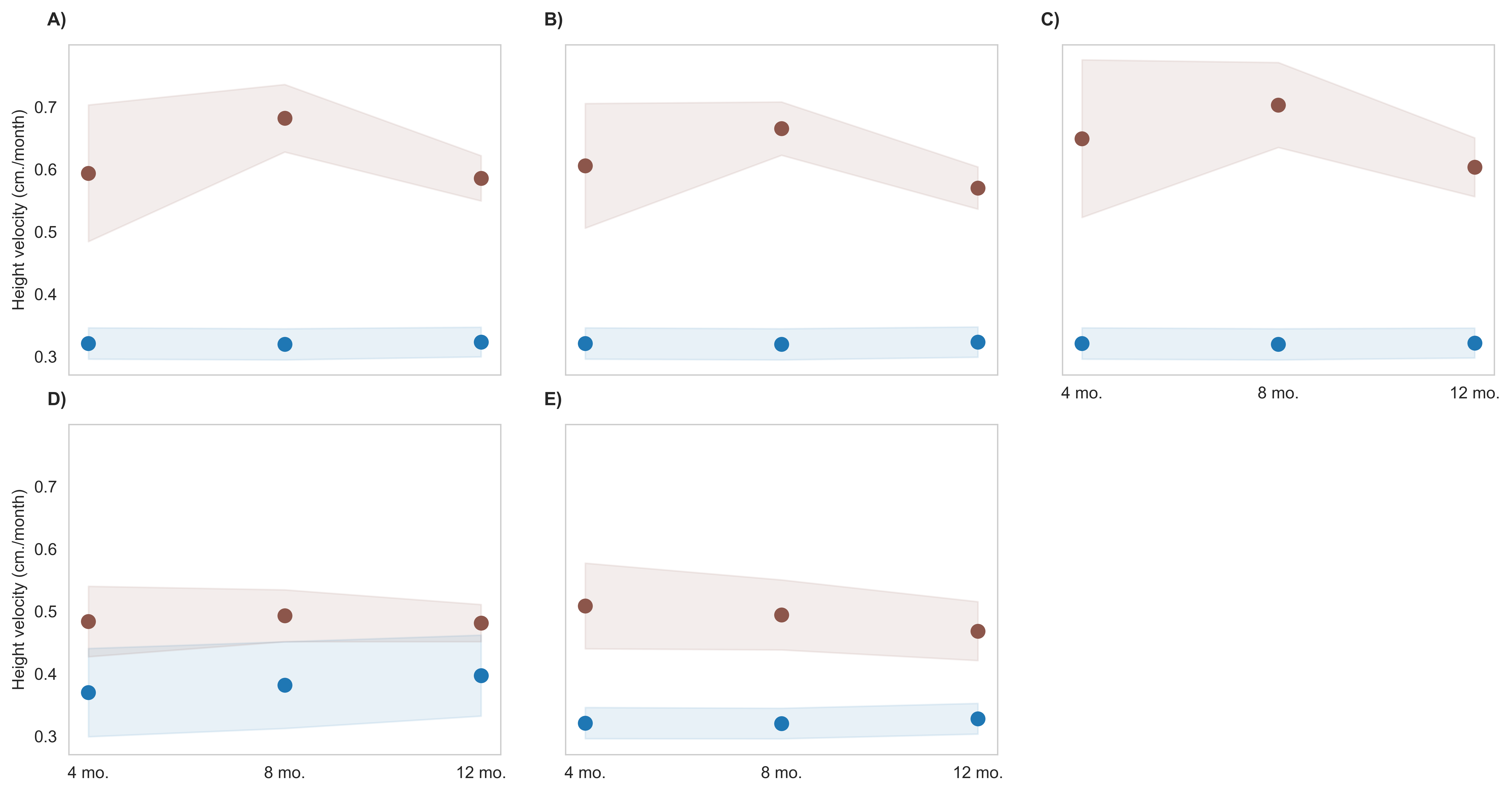

Supplement: S16 Fig — (TIFF) [file pone.0318629.s020.tiff]

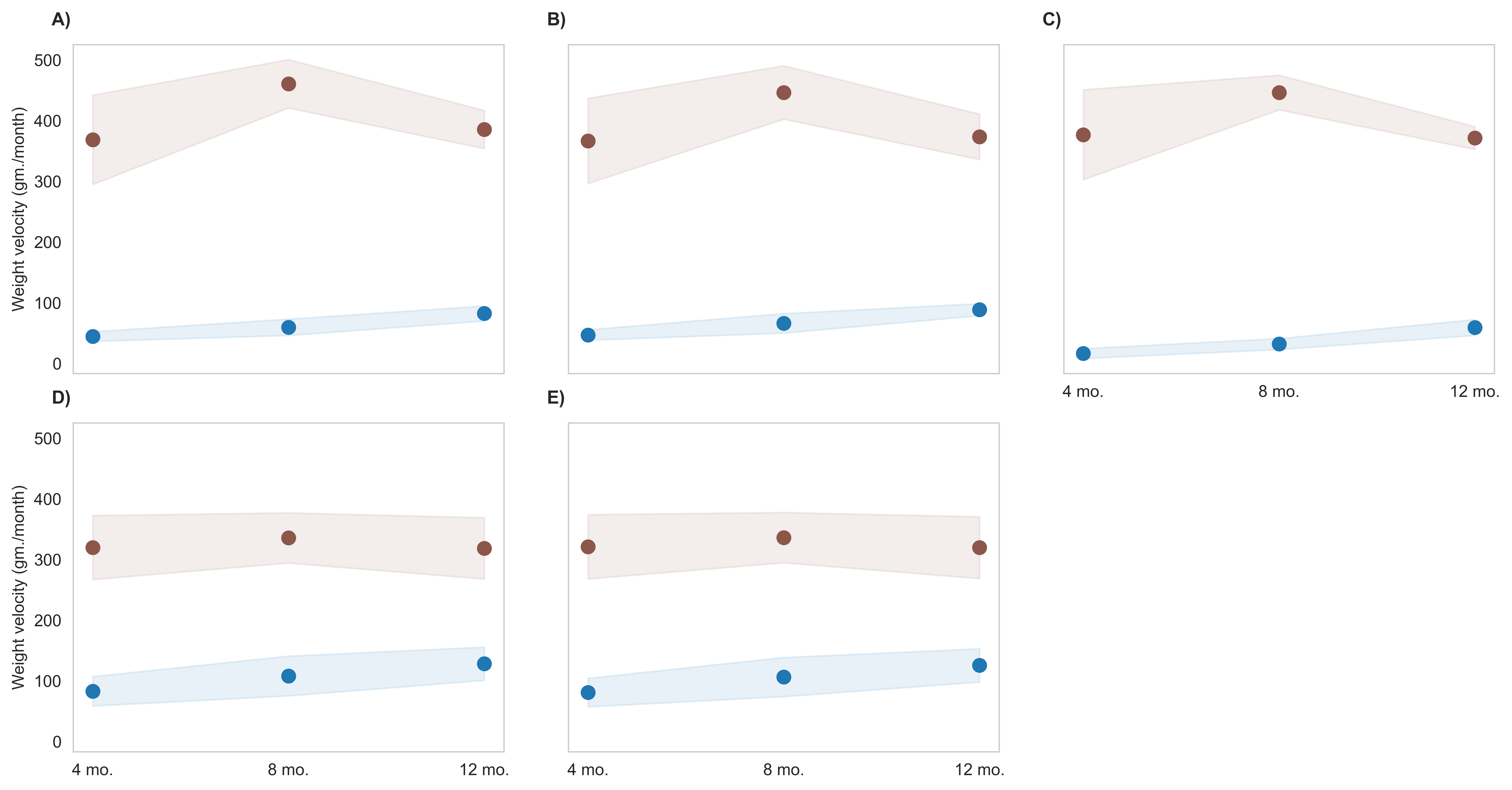

Supplement: S17 Fig — (TIFF) [file pone.0318629.s021.tiff]

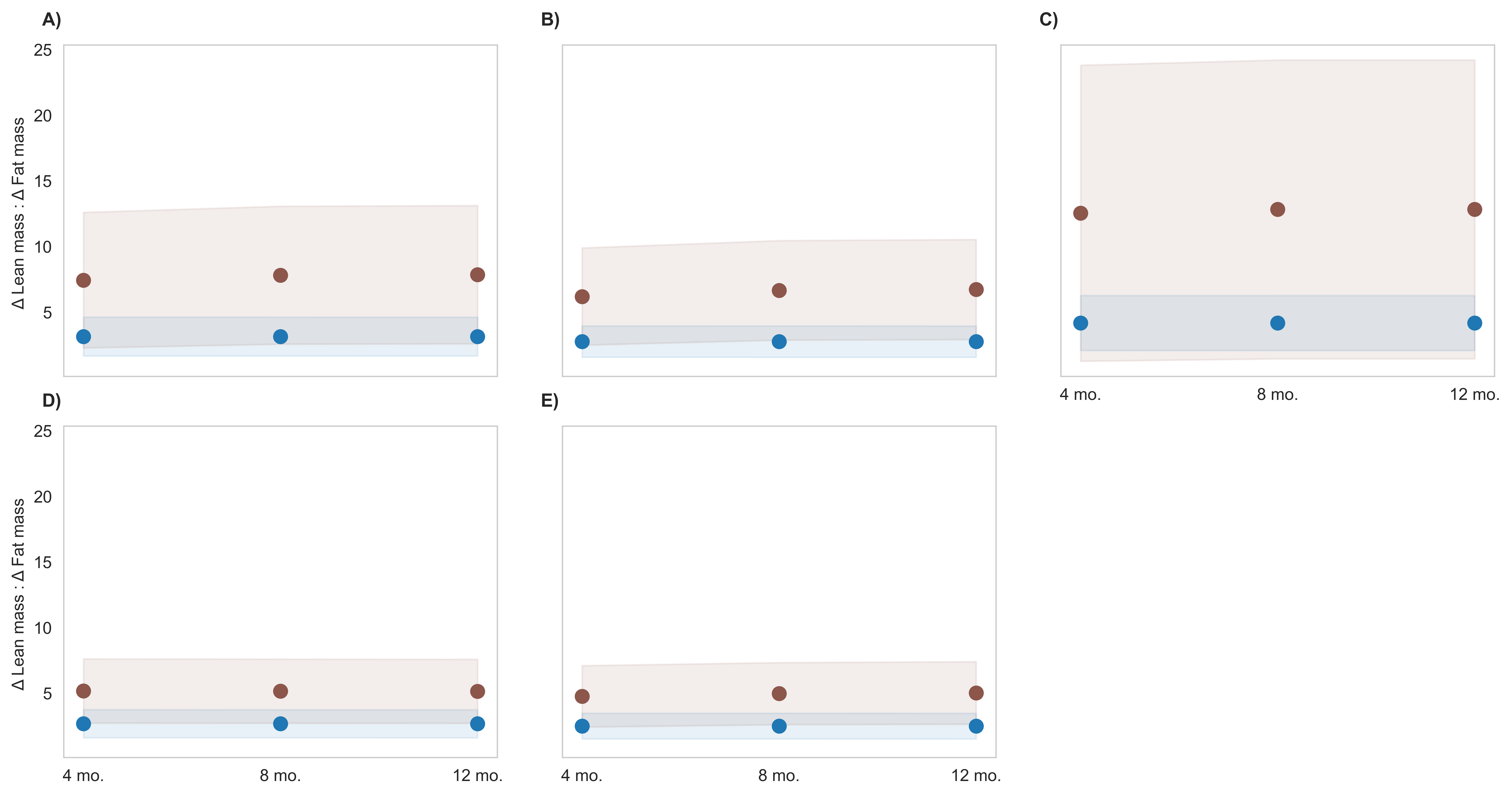

Supplement: S18 Fig — (TIFF) [file pone.0318629.s022.tiff]

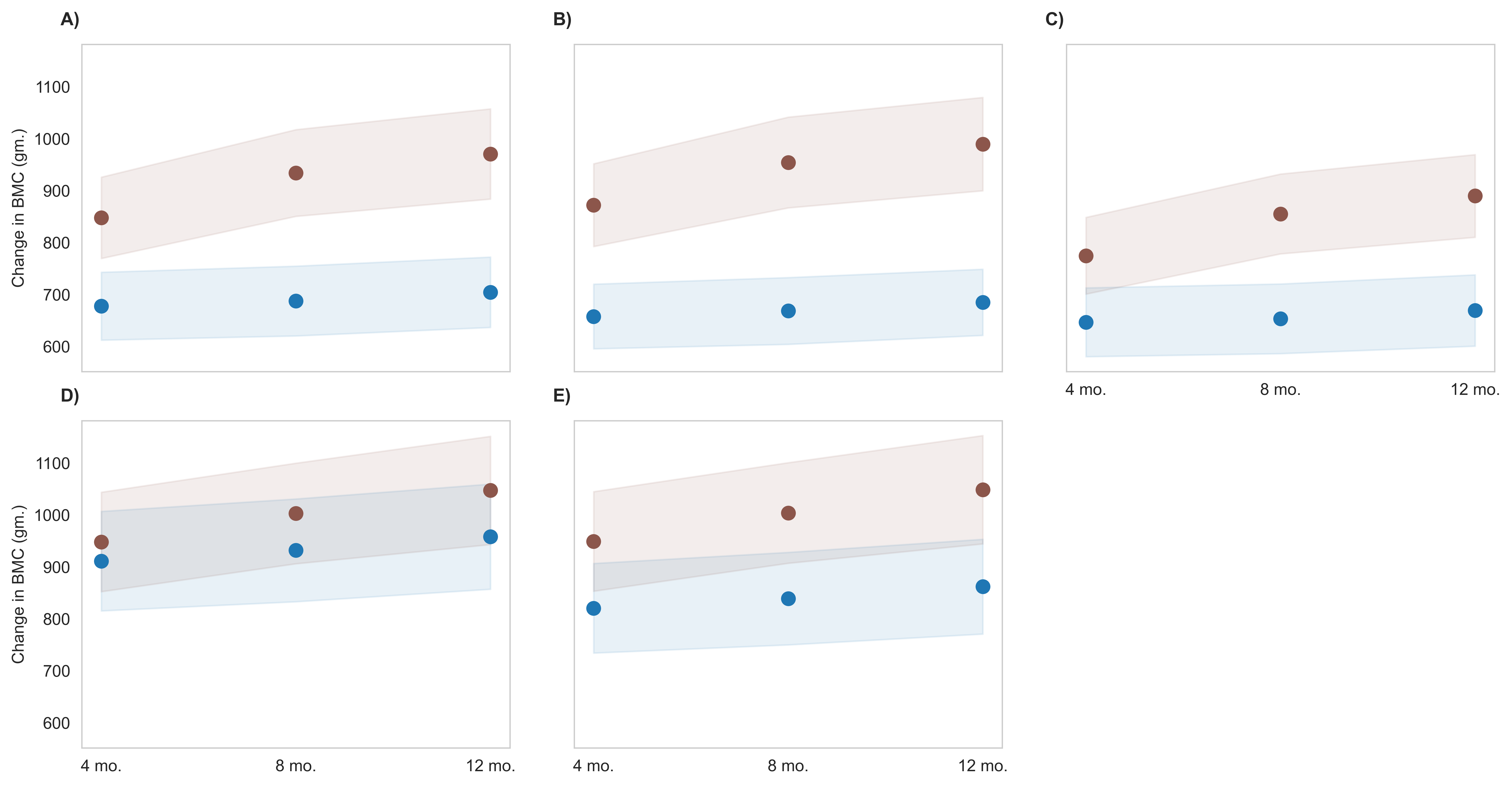

Supplement: S19 Fig — (TIFF) [file pone.0318629.s023.tiff]
